# Construction of a high-density genetic map for grape using specific length amplified fragment (SLAF) sequencing

**DOI:** 10.1371/journal.pone.0181728

**Published:** 2017-07-26

**Authors:** Jiahui Wang, Kai Su, Yinshan Guo, Huiyang Xing, Yuhui Zhao, Zhendong Liu, Kun Li, Xiuwu Guo

**Affiliations:** College of Horticulture, Shenyang Agricultural University, Shenyang, Liaoning, P.R. China; CIRAD, FRANCE

## Abstract

Genetic maps are important tools in plant genomics and breeding. We report a large-scale discovery of single nucleotide polymorphisms (SNPs) using the specific length amplified fragment sequencing (SLAF-seq) technique for the construction of high-density genetic maps for two elite wine grape cultivars, ‘Chardonnay’ and ‘Beibinghong’, and their 130 F_1_ plants. A total of 372.53 M paired-end reads were obtained after preprocessing. The average sequencing depth was 33.81 for ‘Chardonnay’ (the female parent), 48.20 for ‘Beibinghong’ (the male parent), and 12.66 for the F_1_ offspring. We detected 202,349 high-quality SLAFs of which 144,972 were polymorphic; 10,042 SNPs were used to construct a genetic map that spanned 1,969.95 cM, with an average genetic distance of 0.23 cM between adjacent markers. This genetic map contains the largest molecular marker number of the grape maps so far reported. We thus demonstrate that SLAF-seq is a promising strategy for the construction of high-density genetic maps; the map that we report here is a good potential resource for QTL mapping of genes linked to major economic and agronomic traits, map-based cloning, and marker-assisted selection of grape.

## Introduction

Grape (*Vitis vinifera* L., 2*n* = 38) is one of the most important perennial fruit vines worldwide, with a production of 74 million tons over a harvested area of 7 million ha in 2014 (FAO, http://faostat3.fao.org/browse/Q/QC/E). The consumption of table grapes and/or wine has proven to be greatly beneficial to human health [1–4], and the demand for high-quality grapes has increased considerably in recent years. However, grape growth, yield, and quality are affected by various biotic and abiotic stresses. Therefore, for grape breeders, it is important to identify methods for improving the quality characteristics and stress resistance of cultivated grapes. This optimization can be achieved by crossing different germplasms from domesticated or wild-type grapes that possess the desired superior traits [5]. However, the generation using conventional breeding methods of grape cultivars with the preferred traits requires considerable time and can even take decades. Thus, alternative methods are necessary to facilitate the rapid incorporation of these desirable traits in cultivars for large-scale production.

One such method involves the use of genetic maps; these provide a basis for QTL mapping, identification of functional genes, and marker-assisted selection. Genetic linkage maps, particularly high-density genetic maps, are one of the most valuable tools for QTL mapping and high-throughput superior trait selection among various germplasms, including plants and animals. They thus constitute an important means to identify and cultivate resistant, economically viable cultivars, and the construction of such maps is therefore important for grape breeding. Over the past two decades, several unsaturated grape genetic maps have been constructed based on DNA markers, such as randomly amplified polymorphic DNA (RAPD) [6,7], amplified fragment length polymorphism (AFLP) [8], sequence related amplified polymorphism (SRAP) [9], and simple sequence repeat (SSR) [10–12]. However, the application of RAPD, AFLP, and SRAP markers has thus far been limited owing to their dominant inheritance and low transferability. On the contrary, SSR markers have advantages such as co-dominant inheritance, reproducibility, and locus specificity for genetic map construction. However, the number of these markers is generally limited and some of the markers have no sequence information. The development of next-generation sequencing (NGS) technologies and the availability of the full grape genome sequence [13] have facilitated considerable development of single nucleotide polymorphism (SNP) markers [14]. SNPs are the most abundant and stable type of genetic variations in genomes and therefore play an important role in genetic map construction [15,16]. Several efficient NGS-based methods have been used to identify SNPs, such as restriction site associated DNA sequencing (RAD-seq) [17], 2b-RAD [18], double digest RAD (ddRAD) [19], genotyping-by-sequencing (GBS) [20], and specific length amplified fragment sequencing (SLAF-seq) [21]. RAD genotyping is measured by randomly digesting genomic DNA with restriction enzymes; 2b-RAD and ddRAD are derived methods of RAD where 2b-RAD is based on the use of type IIB restriction enzymes and ddRAD is a double-digest technology with two restriction enzymes. Methylation sensitive restriction endonuclease ApeKI is used to digest the genomic DNA in GBS, and SLAF is measured by sequencing the paired-ends of the sequence-specific restriction fragment length. SLAF-seq represents a high-resolution strategy for large-scale *de novo* SNP discovery and genotyping, and this approach has been successfully used to construct high-density genetic maps for many plant and animal species [22–26]. SLAF-seq is a powerful high-throughput technique for rapid and efficient development of SNP markers for genetic map construction.

In the present study, we employed two grape cultivars, ‘Chardonnay’ and ‘Beibinghong’. ‘Chardonnay’, which was used as the female parent, is a grape with thin, yellow-green skin, mainly used for brewing white wine and champagne, and lacking high disease and pest resistance. The male parent, the *V*. *amurensis*-derived ‘Beibinghong’ with thick, dark blue skin, was first bred in northeast China and used for ice red wine production [27]. Moreover, it has high cold tolerance and disease resistance and can overwinter without soil covering in Ji'an, Jilin, China. ‘Chardonnay’ and ‘Beibinghong’ thus exhibit significant differences in several traits, such as skin color and thickness, fruit aroma, and resistance to diseases, and meet the requirements for mapping populations for high-density genetic map construction.

In this study, SLAF-seq was used for rapid discovery of SNPs in F_1_ populations derived from a cross between the two wine grape cultivars. Subsequently, a high-density genetic map of grape was constructed and its characteristics were analyzed in detail. This map is a good potential resource for genetic or QTL mapping of major economic and agronomic traits, map-based cloning, and marker-assisted selection of grape varieties.

## Materials and methods

### Plant material and DNA extraction

A grape hybrid population derived from a cross of ‘Chardonnay’ (*V*. *vinifera*) and ‘Beibinghong’ (*V*. *amurensi*s × *V*. *vinifera*) was generated in May of 2014. The female parent of ‘Beibinghong’ was ‘Zuoyouhong’ which was derived from the cross between F_1_ of ‘Zuoshaner’ (*V*. *amurensis*) × ‘Muscat Rouge’ (*V*. *vinifera*) and ‘74-1-326’ (*V*. *amurensis*). The male parent of ‘Beibinghong’ was ‘86-24-53’ which was derived from the cross between F_1_ of ‘73040’ (*V*. *amurensis*) × ‘Ugni Blanc’ (*V*. *vinifera*) and ‘Shuangfeng’ (*V*. *amurensis*). A total of 331 individuals were produced, of which 130 individuals and their parents were used as the mapping population. The seedlings of the progeny and the parents were planted in the experimental orchard of Shenyang Agriculture University in Shenyang, Liaoning Province, China.

Healthy young leaves were harvested from both parents and each individual F_1_ plant. The samples were immediately stored in liquid nitrogen and transferred to a -80°C freezer. Genomic DNA was extracted using the cetyltrimethylammonium bromide (CTAB) method [28]. DNA concentration was measured using a NanoDrop spectrophotometer (ND2000; Thermo Fisher Scientific, USA) and DNA quality was determined by electrophoresis on 0.8% agarose gels.

### SLAF library construction and high-throughput sequencing

We used an improved SLAF-seq strategy [29]. Two enzymes, *Rsa*I and *Hae*III (New England Biolabs, USA), were used to digest the genomic DNA of each sample after marker discovery and SLAF pilot experiments. A single nucleotide (A) overhang was added subsequently to the digested fragments, and duplex tag-labeled sequencing adapters (PAGE-purified, Life Technologies, USA) were ligated to the A-tailed fragments. Polymerase chain reaction (PCR) was performed using diluted restriction-ligation DNA samples, dNTP, Q5^®^High-Fidelity DNA Polymerase, and PCR primers. The PCR products were then purified and pooled, and pooled samples were separated by 2% agarose gel electrophoresis. Fragments ranging from 400 to 450 bp (with indexes and adaptors) in size were excised and purified using a QIAquick gel extraction kit (Qiagen, Hilden, Germany). Gel-purified products were then diluted, and pair-end sequencing (each end 125 bp) was performed on an Illumina HiSeq 2500 system (Illumina, Inc., San Diego, CA, USA) according to the manufacturer’s recommendations.

### Sequence data grouping and genotyping

Marker identification and genotyping were performed using procedures described by Sun et al. [21]. Briefly, low-quality reads (quality score < 30) were filtered out and then raw reads were sorted to each progeny. Clean reads from the same sample were mapped onto the PN40024 grape genome sequence [30] using BWA (0.7.10-r789) software [31] with parameters set to Score (missed match) = 3, Score (opening gap) = 11, and Score (gap extension) = 4. Sequences mapped to the same position were defined as a single SLAF locus with the depth and integrity thresholds of 7 and 0.3, respectively. Only SLAFs with two to four alleles were identified as polymorphic SLAFs. Genome Analysis Toolkit (GATK) [32] and Sequence Alignment/Map tools (SAMtools) [33] were used to identify SNP loci after local realignment with GATK. Considering the data accuracy, the intersection of SNP calls made by the two tools was regarded as the candidate SNP dataset, and only biallelic SNPs were retained as the final SNP dataset. Polymorphic markers were classified into four segregation patterns (hk × hk, lm × ll, nn × np, and aa × bb). Based on the population type of F_1_, three segregation patterns (excluding aa × bb) were selected for genetic map construction. Genotype scoring was then performed using a Bayesian approach to further ensure the genotyping quality [21]. High-quality SNP markers for genetic mapping were filtered and those with the average sequence depths of >40-fold in the parents and with less than 5% missing data were retained. The chi-square test was then performed to examine the segregation distortion, and markers with significant segregation distortion (P < 0.05) were initially excluded from map construction and added later as accessory markers.

### Linkage map construction

SNP markers were partitioned primarily into linkage groups (LGs) based on their locations on the grape genome. Next, the modified logarithm of odds (MLOD) scores between markers was calculated to further confirm the robustness of markers for each LG. Markers with MLOD scores < 5 were filtered prior to ordering. A HighMap strategy was applied to order the SNP markers and correct genotyping errors within LGs [34]. Briefly, recombinant frequencies and LOD scores were calculated using a two-point analysis and these were applied to infer linkage phases. Then, enhanced Gibbs sampling, spatial sampling, and simulated annealing algorithms were combined to conduct an iterative process of marker ordering [35,36]. The mapping algorithm was repeated until all markers were mapped appropriately. The error correction strategy of SMOOTH was then applied based on the parental contribution of genotypes [37], and a k-nearest neighbor algorithm was applied to impute missing genotypes [38]. Skewed markers were then incorporated into this map using the multipoint maximum likelihood method. Map distances were estimated using the Kosambi mapping function [39]. The collinearity between the genetic and physical positions, the haplotype map, and the heat map were used to evaluate the quality of the constructed linkage map. The methods were conducted as described by Liu et al. [40].

## Results

### Analysis of SLAF-seq data and markers

**Fig 1:**
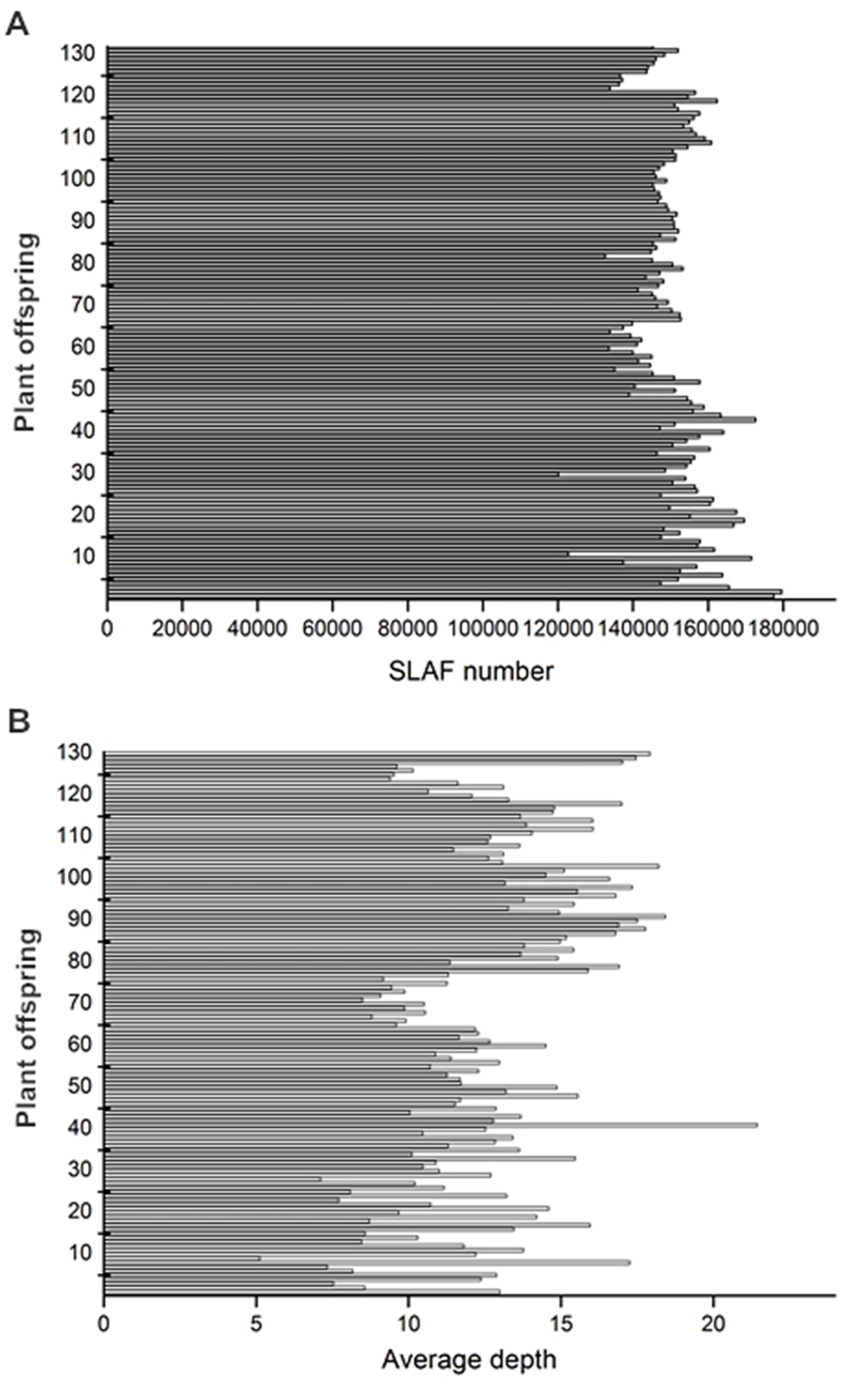
Number of SLAFs (A) and average sequencing depths (B) of F_1_ population. The x-axis indicates the number of SLAFs (A) and the average depths (B); the y-axis indicates individual F_1_ offspring.

A total of 372.53 M paired-end reads were generated for this grapevine population; of those 91.92% were high quality, which corresponds to a quality score of at least 30 (Q30). The average guanine-cytosine (GC) content was 40.03%. The reads number in female and male parents was 11,355,661 and 9,702,794, respectively. On average, 2,767,511 reads per individual were generated (Table 1). Of a total of 202,349 high-quality SLAFs, 179,508 were detected in the female parent and 177,451 in the male parent; the average sequence depth of each SLAF from the parents was 33.81-fold and 48.20-fold for the female and male parents, respectively. The analysis of the mapping population revealed that 149,910 SLAFs were generated, and the average depth of each SLAF was 12.66-fold for each offspring (Fig 1).

**Table 1:** Summary of SLAF-seq data for grape.

Samples	Total reads	SLAFs number	Total depth	Average depth
Female	11,355,661	179,508	6,069,279	33.81
Male	9,702,794	177,451	8,553,213	48.20
Offspring	2,767,511	149,910	1,906,168	12.66

Of these high-quality SLAFs, 181,337 were mapped onto the grape genome sequence and 144,972 were polymorphic with a polymorphism rate of 71.64%. A total of 1,762,745 SNPs were obtained. The number of SLAFs and SNPs in each chromosome differed; the number of SLAFs ranged from 7,601 in chromosome 17 to 12,883 in chromosome 18, while the number of SNPs ranged from 73,727 in chromosome 17 to 119,963 in chromosome 18 (Table 2). Of the 1,762,745 polymorphic SNPs, 99,634 were biallelic and classified into four segregation patterns—aa × bb (22,460), hk × hk (7,240), lm × ll (40,618), and nn × np (29,316). Besides the aa × bb genotype, the other three patterns were used for genetic map construction, and a total of 77,174 SNPs fell into these classes (4.38% of total polymorphic SNPs).

**Table 2:** Distribution of SLAFs and SNPs on chromosomes.

Linkage groups ID	SLAFs number	SNPs number
LG1	10,019	93,391
LG2	8,426	78,090
LG3	8,815	79,777
LG4	10,476	97,241
LG5	11,315	107,752
LG6	8,682	78,428
LG7	8,503	78,629
LG8	10,157	101,232
LG9	8,438	92,920
LG10	7,972	77,727
LG11	8,853	80,038
LG12	9,588	97,777
LG13	10,591	100,828
LG14	12,430	123,501
LG15	8,808	91,325
LG16	8,656	85,805
LG17	7,601	73,727
LG18	12,883	119,963
LG19	9,724	104,594
Total	181,337	1,762,745

### Characteristics of the genetic maps

**Fig 2:**
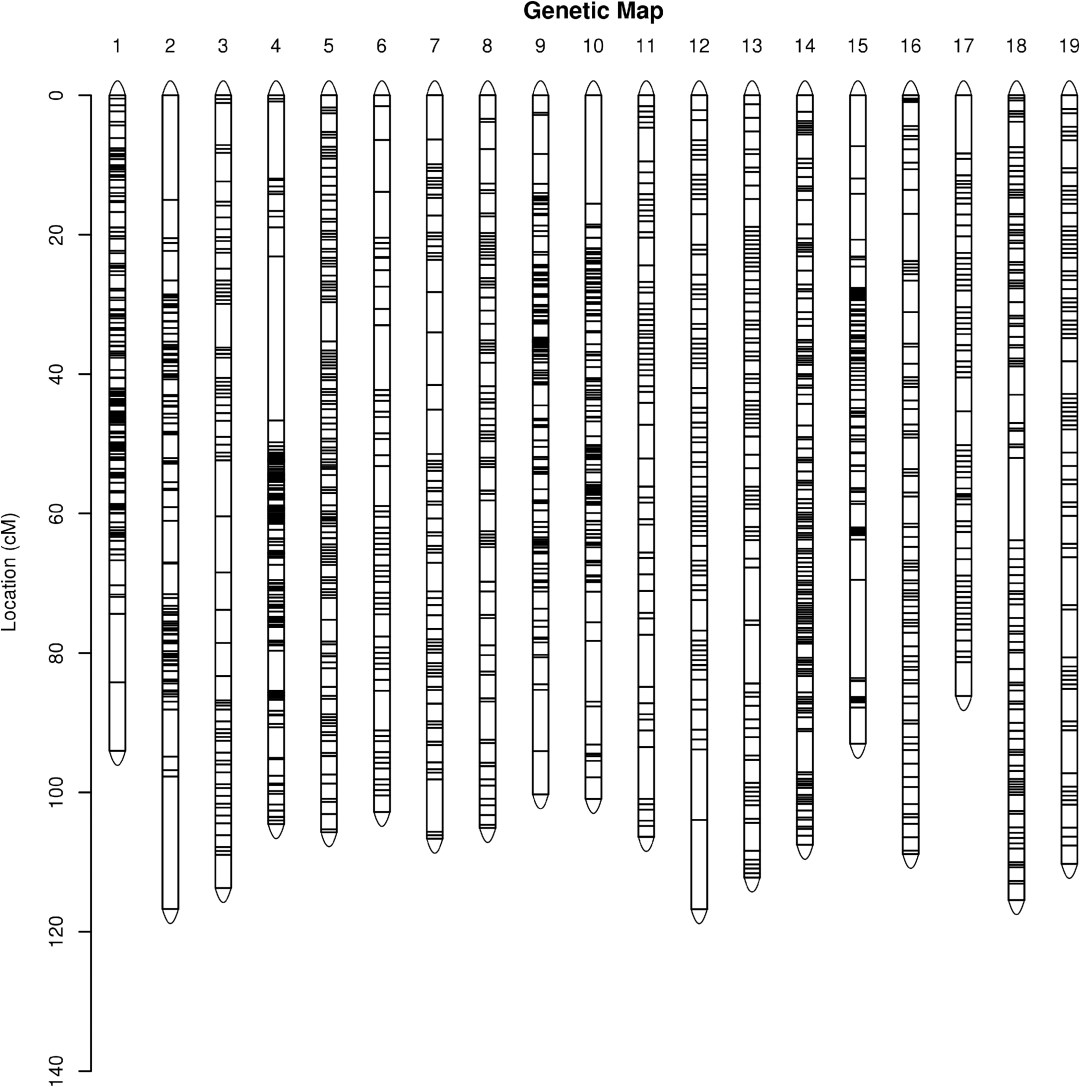
Genetic map lengths and marker distribution in 19 linkage groups of the female parent. Genetic distance is indicated by the vertical scale in centimorgans (cM). Black lines represent mapped markers. 1–19 represent corresponding linkage groups ID.

All mapped markers fell into 19 LGs based on the chromosome numbers. There were 6,002 markers in the female map with a total length of 2,186.38 cM (Fig 2). The genetic length of each LG ranged from 86.18 cM (LG17) to 237.19 cM (LG18). LG14 contained the largest number of markers (518), with the average marker distance being 0.21 cM, whereas LG2 had the lowest marker number (163), with an average marker distance of 0.72 cM (Table 3). The percentage of ‘Gap ≤ 5’ (gaps wherein the distance between adjacent markers was smaller than 5 cM) for each LG ranged from 96.91% (LG2) to 99.81% (LG14) (Table 4).

**Table 3:** The genetic length and markers number of 19 linkage groups.

Linkage groups ID	No of SNP markers			Genetic length (cM)			Physical length ^a^ (bp)
	Female map	Male map	Integrated map	Female map	Male map	Integrated map	
LG1	296	356	617	94.06	118.01	113.06	23037639
LG2	163	40	182	116.74	116.13	110.75	18779844
LG3	281	295	521	113.70	102.10	104.10	19341862
LG4	322	355	581	104.56	74.05	77.21	23867706
LG5	438	488	872	105.72	110.61	104.02	25021643
LG6	274	307	530	102.81	119.15	116.06	21508407
LG7	336	303	593	106.64	109.44	108.27	21026613
LG8	342	217	556	105.11	105.76	105.94	22385789
LG9	254	147	387	100.28	113.01	112.67	23006712
LG10	230	156	349	100.95	92.17	101.83	18140952
LG11	322	299	581	106.39	112.03	103.09	19818926
LG12	375	267	611	116.76	107.14	115.87	22702307
LG13	383	348	666	112.22	107.44	102.07	24396255
LG14	518	241	747	107.55	102.52	103.55	30274277
LG15	175	50	221	93.02	96.14	99.55	20304914
LG16	191	196	347	108.84	75.38	71.42	22053297
LG17	315	176	445	86.18	91.19	94.54	17126926
LG18	432	320	711	237.19	107.57	116.15	29360087
LG19	355	179	525	167.66	105.12	109.81	24021853
Total	6,002	4,740	10,042	2186.38	1964.96	1969.95	426176009

^a^ Physical size is according to Jaillon et al. [30].

**Table 4:** The markers spacing and coverage of 19 linkage groups.

Linkage groups ID	Average spacing (cM)			Gaps≤5 (Max Gap)			Coverage ^b^ (%)		
	Female map	Male map	Integrated map	Female map	Male map	Integrated map	Female map	Male map	Integrated map
LG1	0.32	0.33	0.18	99.32% (9.84)	98.59% (12.22)	99.68% (11.81)	99.04	99.72	99.72
LG2	0.72	2.90	0.61	96.91% (19.00)	79.49% (13.30)	98.34% (10.52)	92.51	94.12	94.12
LG3	0.40	0.35	0.20	98.21% (8.04)	100.00% (4.84)	99.81% (5.02)	99.16	99.58	99.58
LG4	0.32	0.21	0.13	99.07% (23.52)	99.72% (5.93)	100.00% (4.50)	99.84	99.43	99.84
LG5	0.24	0.23	0.12	99.77% (5.60)	100.00% (4.83)	100.00% (2.36)	99.84	99.70	99.84
LG6	0.38	0.39	0.22	98.17% (9.27)	99.35% (20.66)	100.00% (4.84)	99.47	98.70	99.47
LG7	0.32	0.36	0.18	98.51% (7.55)	98.34% (9.92)	100.00% (4.96)	99.68	99.92	99.92
LG8	0.31	0.49	0.19	99.71% (5.49)	98.61% (18.39)	99.82% (5.42)	99.68	99.50	99.68
LG9	0.39	0.77	0.29	98.81% (8.80)	96.58% (22.66)	99.22% (9.78)	99.79	98.43	99.79
LG10	0.44	0.59	0.29	98.69% (15.53)	98.71% (11.58)	99.43% (9.57)	99.29	98.79	99.29
LG11	0.33	0.37	0.18	99.38% (7.45)	97.99% (12.93)	99.83% (6.71)	99.89	99.89	99.89
LG12	0.31	0.40	0.19	99.47% (12.81)	98.50% (30.83)	99.67% (6.40)	99.33	98.36	99.33
LG13	0.29	0.31	0.15	99.48% (8.39)	99.14% (8.58)	100.00% (3.57)	91.21	97.05	97.05
LG14	0.21	0.43	0.14	99.81% (5.88)	97.50% (11.82)	100.00% (4.43)	99.53	99.90	99.90
LG15	0.53	1.92	0.45	97.13% (14.10)	81.63% (13.60)	99.09% (6.56)	99.31	99.34	99.34
LG16	0.57	0.38	0.21	99.47% (6.77)	99.49% (6.17)	99.71% (6.13)	98.37	99.73	99.73
LG17	0.27	0.52	0.21	99.68% (8.35)	98.29% (6.57)	99.55% (8.35)	98.02	99.10	99.10
LG18	0.55	0.34	0.16	99.07% (24.28)	98.43% (14.57)	100.00% (3.41)	99.91	99.44	99.91
LG19	0.47	0.59	0.21	98.31% (33.89)	97.19% (10.21)	100.00% (4.41)	97.28	96.35	97.28
Average	0.39	0.62	0.23	98.89%	96.71%	99.69%	98.48	98.79	99.09

^b^ The coverage is calculated as the ratio of the physical distance between the beginning and the end marker and the total physical distance of each linkage group.

**Fig 3:**
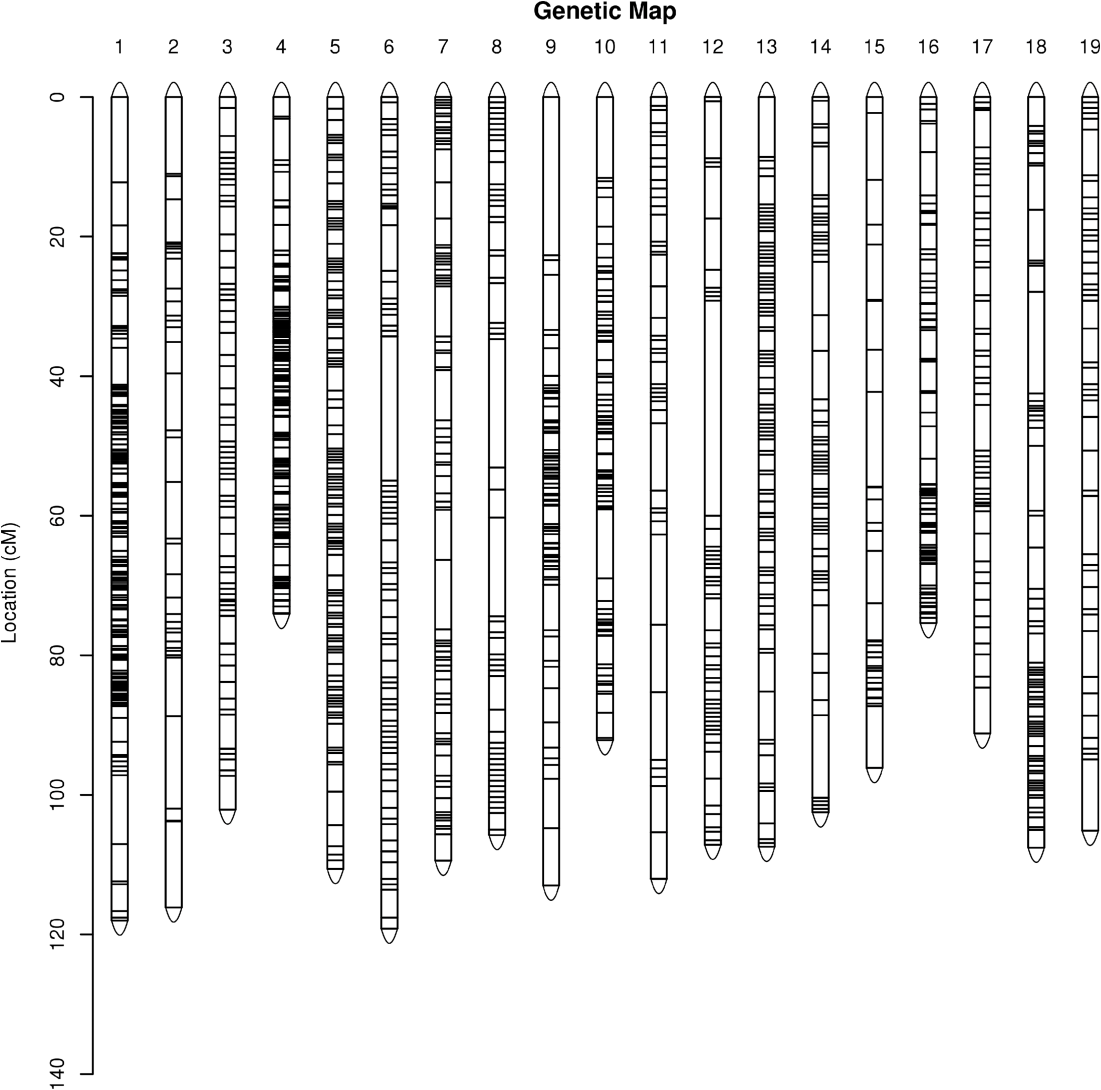
Genetic map lengths and marker distribution in 19 linkage groups of the male parent.

The map of the male parent contained 4,740 markers spanning a total of 1,964.96 cM (Fig 3). LG4 was the shortest LG (length 74.05 cM) and contained 355 markers, with an average genetic distance of 0.21 cM, whereas LG6 was the longest group (length 119.15 cM) and contained 307 markers, with an average genetic distance of 0.39 cM (Table 3). The percentage of ‘Gap ≤ 5’ for each LG ranged from 79.49% (LG2) to 100.00% (LG3 and LG5) (Table 4).

**Fig 4:**
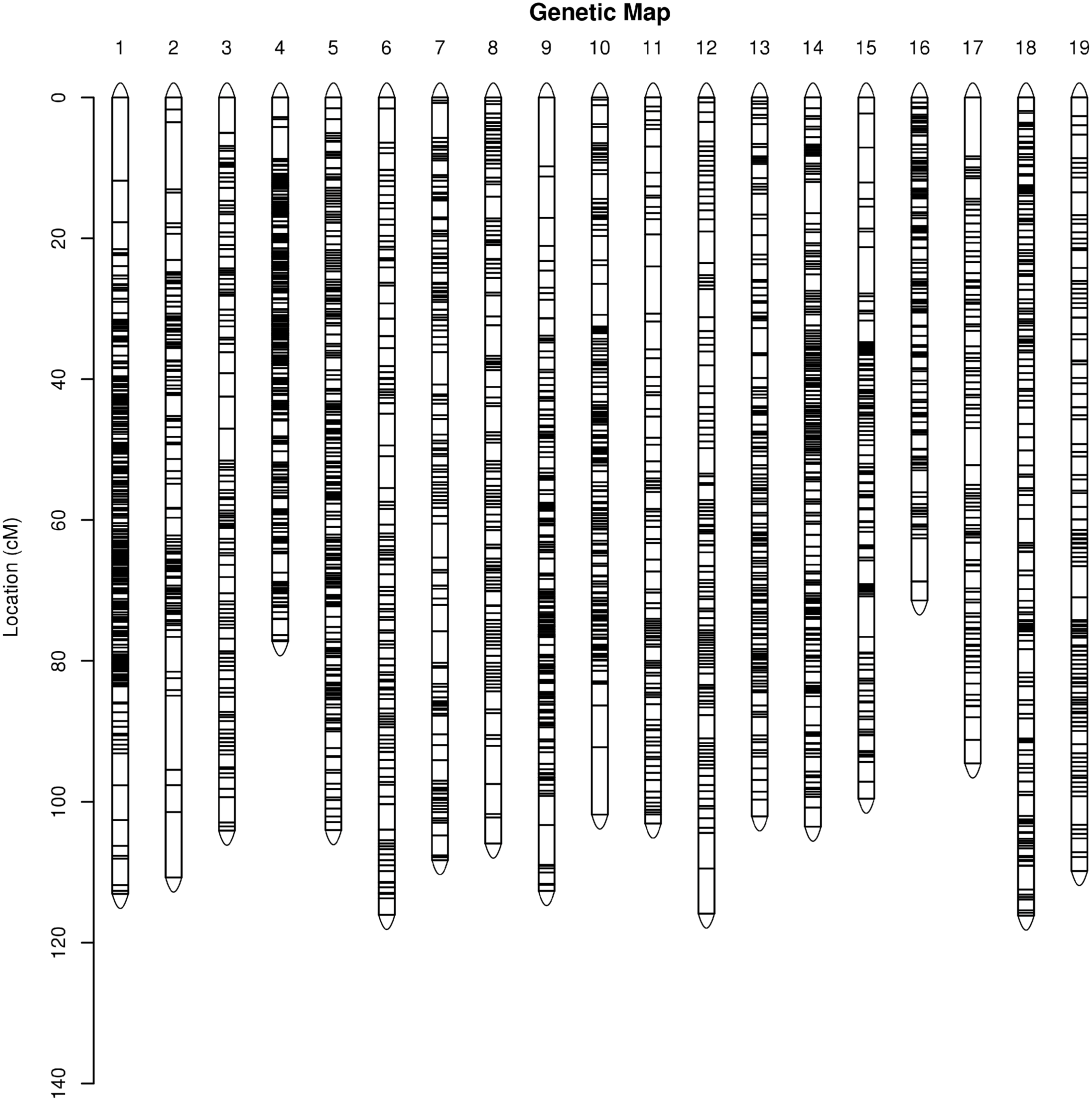
Genetic lengths and marker distribution in 19 linkage groups of the integrated map.

The integrated grape map contained 10,042 markers spanning 1,969.95 cM with an average inter-marker distance of 0.23 cM (Fig 4). These 10,042 markers had an average coverage of 138.03-fold in the parents and 38.76-fold in the F_1_ offspring. The genetic length of the LGs ranged from 77.21 cM (LG4) to 116.15 cM (LG18), with an average length of 103.68 cM. LG5 was the most saturated, spanning 104.02 cM with 872 markers and the average genetic distance of 0.12 cM, whereas LG2 was the least saturated with the length of 110.75 cM and contained the least number of markers (only 182) (Table 3). Moreover, the average percentage of ‘Gap ≤ 5’ was 99.69%. ‘Gap ≤ 5’ was not detected on LG4, LG5, LG6, LG7, LG13, LG14, LG18, and LG19 (Table 4); two gaps larger than 10 cM were located one in LG1 and one in LG2.

### Evaluation of the genetic map

The correlation of genetic and physical positions is an important factor in the quality of a genetic map [41]. The collinearity between the genomic location of mapped SNP markers and physical positions is presented in S1 Fig. The Spearman correlation coefficient in 19 LGs ranged from 0.75 to 0.99, and it was higher than 0.92 in 73.68% of them (Table 5). The results indicated that the correlation of the genetic and physical positions was high in most LGs.

**Table 5:** The Spearman correlation coefficients between the genetic and physical positions of each linkage group on the integrated map.

Linkage group ID	Spearman	Linkage group ID	Spearman
LG1	0.97	LG11	0.96
LG2	0.75	LG12	0.86
LG3	0.93	LG13	0.96
LG4	0.96	LG14	0.92
LG5	0.97	LG15	0.94
LG6	0.99	LG16	0.97
LG7	0.88	LG17	0.91
LG8	0.88	LG18	0.94
LG9	0.84	LG19	0.95
LG10	0.96	Average	0.92

Haplotype and heat maps were also used to evaluate the quality of the genetic map. Haplotype maps can directly reflect recombination events in each individual. The occurrence of double crossovers and deletions is reflected in a haplotype map as genotyping and marker-order errors. Haplotype maps were generated for each F_1_ individual and for the parental controls using 10,042 SNP markers as described by West et al. [42] (S1 File). There was no deletion detected in any LG.

Heat maps were also generated by using pair-wise recombination values for the 10,042 mapped SNP markers (S2 File). Additionally, heat maps can indicate the recombination between markers within one single LG; they could thus be used to identify potential marker ordering errors, pair-wise recombination taking place mainly as a result of hotspot regions for genomic recombination, and sequencing-related genotyping errors. In general, most LGs were determined to perform well.

## Discussion

### SLAF sequencing and large-scale marker development

The SLAF-seq strategy, a combination of locus-specific amplification and high-throughput sequencing, has been subjected to a series of critical trials to verify its high efficiency and accuracy of the generated markers [21]. Recently, the SLAF-seq technology has been used successfully to develop a large number of SLAF markers and for the construction of high-density genetic maps for many plants, including soybean [43], sweet cherry [24], cucumber [44], watermelon [45], red sage [46], and willow [47].

High-throughput sequencing of the SLAF libraries yielded a total of 372.53 M paired-end reads containing 202,349 high-quality SLAFs; the SLAF polymorphism rate was 71.64%. Large-scale SNP markers were developed based on SLAF sequencing data. However, since the presence of some erroneous and missing values in SLAF sequencing data is inevitable, molecular markers must be stringently filtered to avoid false positives [21,48]. Of the 1,762,745 SNPs initially identified, only 77,174 SNPs were considered effective markers for use in the subsequent linkage analysis. These new markers constitute a more effective tool than currently used methods for genetic studies, such as genetic diversity assessment, genetic relationship analysis, and germplasm resource identification [49].

### Construction and importance of grape genetic maps

The development of numerous molecular markers and marker types is a key step for high-density map construction. Several conventional molecular markers, such as RAPDs, AFLPs, SRAPs, and SSRs, were previously widely used to construct grape genetic maps [7–9,11]. Since the construction of the first grape genetic map using RAPD, RFLP, and isozyme markers [6], a number of genetic maps have been developed. However, most current genetic maps contain only a few hundred markers, some of which have no sequence information; LG numbers are also inconsistent in some cases owing to the inefficiency and high genotyping costs of the markers. The saturation, density, and accuracy of currently available genetic maps are thus limited [50]. Therefore, it is necessary to rapidly develop large-scale molecular markers for the construction of high-density grape genetic maps.

The development of NGS technology permits the identification of millions of SNP markers across the genome. This technology has been widely used for large-scale genotyping and high-density genetic map construction in several studies. RAD sequencing has been successfully used to construct high-density genetic maps for ryegrass [51], globe artichoke [52], and eggplant [53]; several maps have been constructed using 2b-RAD sequencing of rice [54], *Brachypodium distachyon* [55], and bighead carp [56]; and genetic maps for strawberry [57], peanut [58], and lotus [59] have been constructed by ddRAD sequencing. The GBS approach has been used for the construction of genetic maps of apple [60], barley, and wheat [61]. Recent years have also seen important research progress being made with respect to the construction of high-density genetic maps for grape using NGS technology. Wang et al. [5] constructed a genetic map with 1,646 SNPs and a length of 1,917.13 cM using RAD sequencing. Genetic maps were constructed based on GBS for *V*. *rupestris* ‘B38’ (1,146 SNPs) and ‘Chardonnay’ (1,215 SNPs), spanning 1,645 cM and 1,967 cM, respectively [62]. Guo et al. [63] successfully constructed a genetic map with a length of 1929.13 cM containing 7,199 markers using SLAF sequencing. Genetic maps were also obtained from Illumina chips. Using the 18 K Infinium chip, Houel et al. [64] reported genetic maps for ‘Picovine’ (408 SNPs) and ‘Ugni Blanc’ (714 SNPs), spanning 606 cM and 1,301 cM, respectively. In the present study, we constructed a high-density genetic map of grape with a total genetic distance of 1,969.95 cM and 10,042 mapped markers. The cover rate of the map length was 99.09%, and the average inter-marker distance was 0.23 cM; the number of mapped markers, average genetic distance, and genome cover rate of the currently reported map present considerable improvements on previously published genetic maps for grape.

Herein, a newly developed HighMap strategy with an iterative process of marker ordering and error genotype correction was applied to construct genetic maps using individual markers; for bin mapping, markers were assigned to bins. A “bin” is a group of markers with a unique segregation pattern and is separated from adjacent bins by a single recombination event. The bin strategy reduces the utilization of genotyping data and recombination information [34]. Therefore, HighMap software may construct a map with higher possible average density depending on the same population size.

Despite these advantages, the constructed map also contained defects such as the presence of several large gaps in sections of the LGs and the weak collinearity of individual LGs. Despite the average distance between adjacent markers on the map being very short (only 0.23 cM), two gaps larger than 10 cM were detected in LG1 and LG2. These large gaps may be due to the absence of marker polymorphism and limited marker detection in these regions [21,65]. Most of the LGs showed good correlation between the genetic and physical positions, but there were also rearrangements in some chromosome regions. Among the 19 LGs, LG2, LG7, LG8, LG9, and LG12 had a lower collinearity compared to other LGs. Imperfect genome assembly, mapping population number, and assembly errors might be common reasons for this inconsistency [66,67]. Moreover, since the male parent ‘Beibinghong’ is the results of interspecies cross (*V*. *amurensis* × *V*. *vinifera*), some of the regions might originate from *V*. *amurensis* and differ from the reference genome (*V*. *vinifera*). Therefore, the noncollinearity observed in some chromosome regions might indicate the presence of some variations among different grape species that were developed during the course of evolution. We found that many traits in the 130 progenies that bloomed and fruited in 2016 were segregated. In the future, we intend to increase the mapping population size in order to improve map saturation. This improved map will lay the foundation for QTL mapping and identification of candidate genes related to major economic and agronomic traits.

SLAF-seq is a promising rapid and cost-effective strategy for the construction of high-density genetic maps to facilitate the incorporation of desirable traits in cultivated grapes. In this study, 202,349 high-quality SLAFs were developed using the SLAF-seq method. Large-scale SNP markers were developed and used for the construction of a high-density genetic map for grape. A total of 10,042 mapped markers were distributed in 19 LGs. The genetic map spanned 1,969.95 cM with an average inter-marker distance of 0.23 cM. Furthermore, this map will serve as a valuable tool for grape breeders for genetic or QTL mapping or association mapping of important agronomic traits, map-based gene cloning, comparative mapping, and marker-assisted breeding.

## Supporting information

S1 Fig Correlation of genetic and physical positions. The x-axis represents the genetic distance of each linkage group (LG); the y-axis represents the physical position of each LG.
(TIF)

S1 File Haplotype map of the genetic map. Each two columns represent the genotype of an individual. The first column of each individual represents ‘Beibinghong’ (the male parent); the second column of each individual represents ‘Chardonnay’ (the female parent). Rows correspond to genetic markers. Green indicates the first allele from the parent, blue refers to the second allele from the parent, and gray denotes missing data.
(ZIP)

S2 File Heat map of the genetic map. Each cell represents the recombination rate of two markers. Yellow and purple indicate lower and higher recombination rates, respectively. Gray denotes missing data.
(ZIP)

S3 File Marker genotypes used for mapping. “–” represents missing data.
(XLSX)
