# Supplementary figures and images for: Construction of a high-density genetic map for grape using specific length amplified fragment (SLAF) sequencing
Source: PLoS One. 2017 Jul 26;12(7):e0181728. doi: 10.1371/journal.pone.0181728 (PMC5528875; doi:10.1371/journal.pone.0181728)

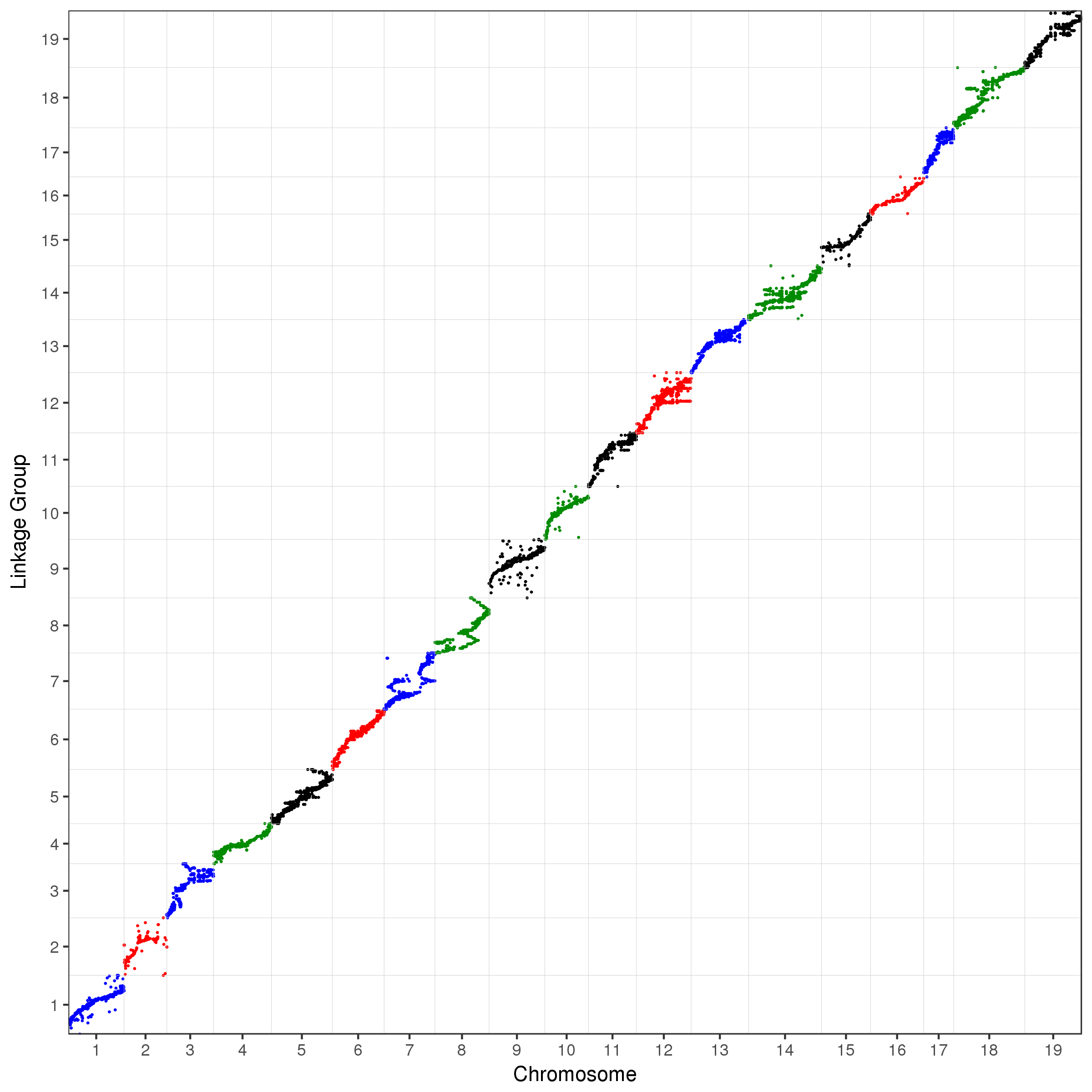

Supplement: S1 Fig — The x-axis represents the genetic distance of each linkage group (LG); the y-axis represents the physical position of each LG. (TIF) [file pone.0181728.s001.tif]

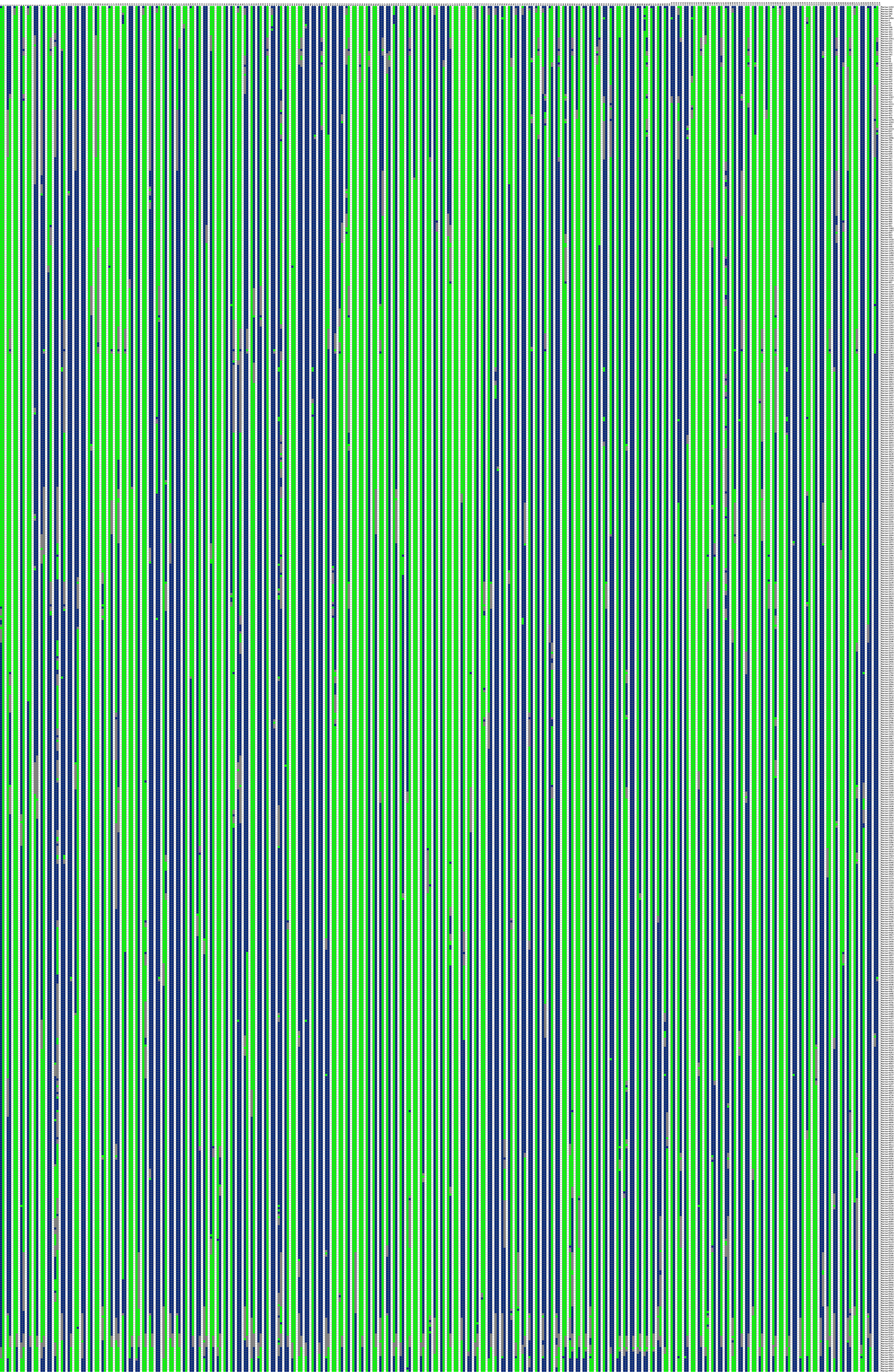

Supplement: S1 File — Each two columns represent the genotype of an individual. The first column of each individual represents ‘Beibinghong’ (the male parent); the second column of each individual represents ‘Chardonnay’ (the female parent). Rows correspond to genetic markers. Green indicates the first allele from the parent, blue refers to the second allele from the parent, and gray denotes missing data. (ZIP) [file pone.0181728.s002.zip › S1_File/chr1.sexAver.repair.tq.png]

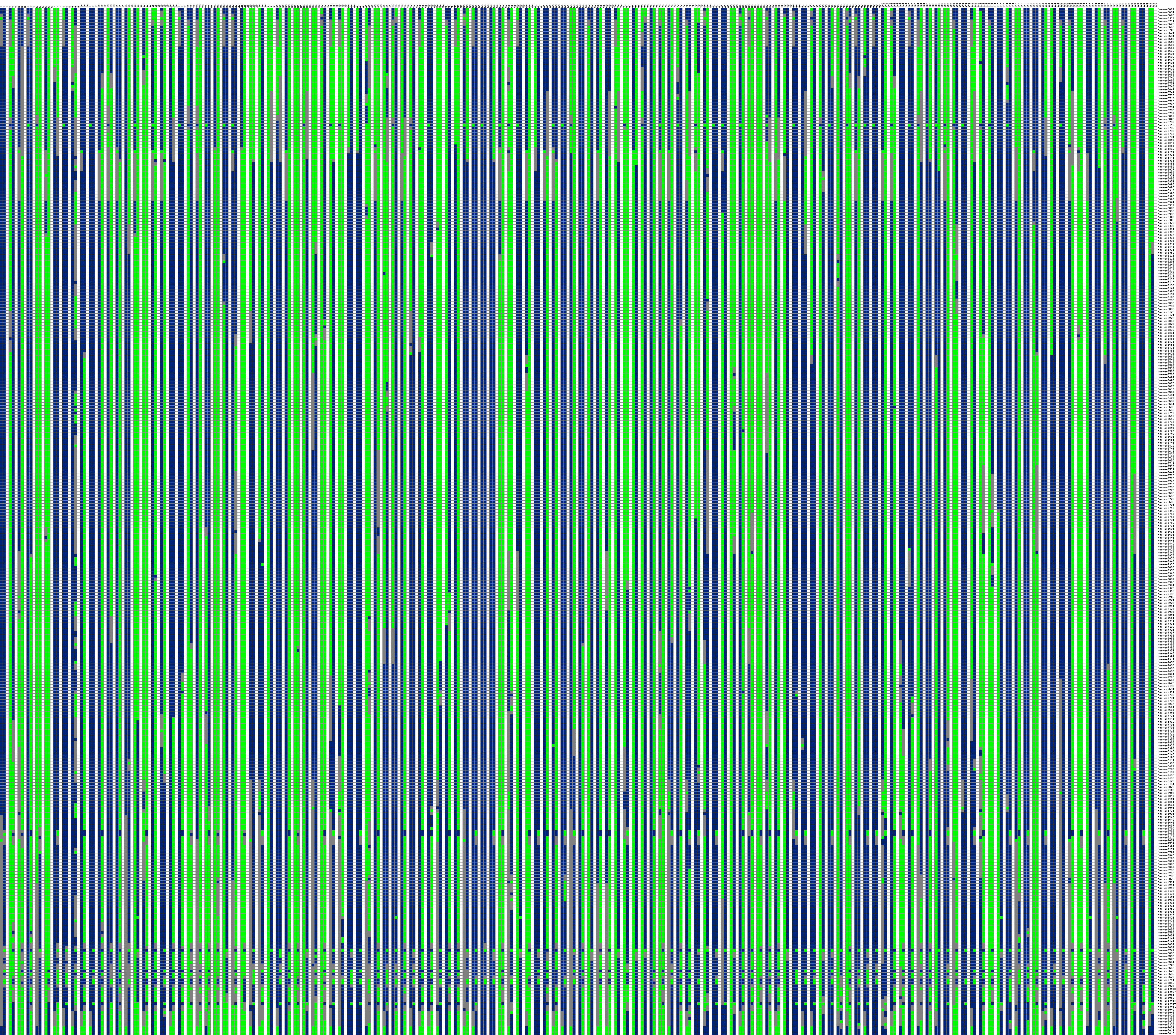

Supplement: S1 File — Each two columns represent the genotype of an individual. The first column of each individual represents ‘Beibinghong’ (the male parent); the second column of each individual represents ‘Chardonnay’ (the female parent). Rows correspond to genetic markers. Green indicates the first allele from the parent, blue refers to the second allele from the parent, and gray denotes missing data. (ZIP) [file pone.0181728.s002.zip › S1_File/chr10.sexAver.repair.tq.png]

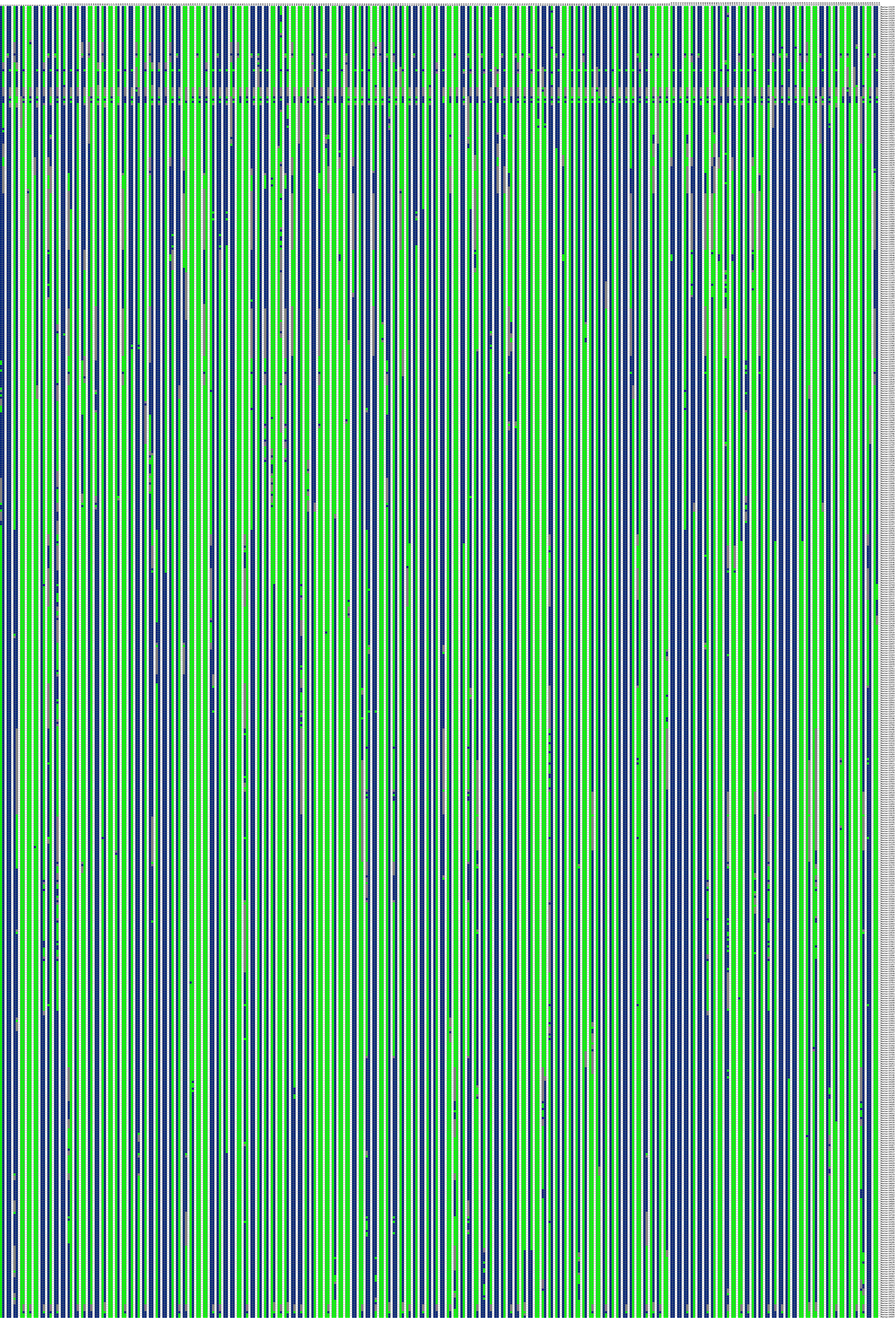

Supplement: S1 File — Each two columns represent the genotype of an individual. The first column of each individual represents ‘Beibinghong’ (the male parent); the second column of each individual represents ‘Chardonnay’ (the female parent). Rows correspond to genetic markers. Green indicates the first allele from the parent, blue refers to the second allele from the parent, and gray denotes missing data. (ZIP) [file pone.0181728.s002.zip › S1_File/chr11.sexAver.repair.tq.png]

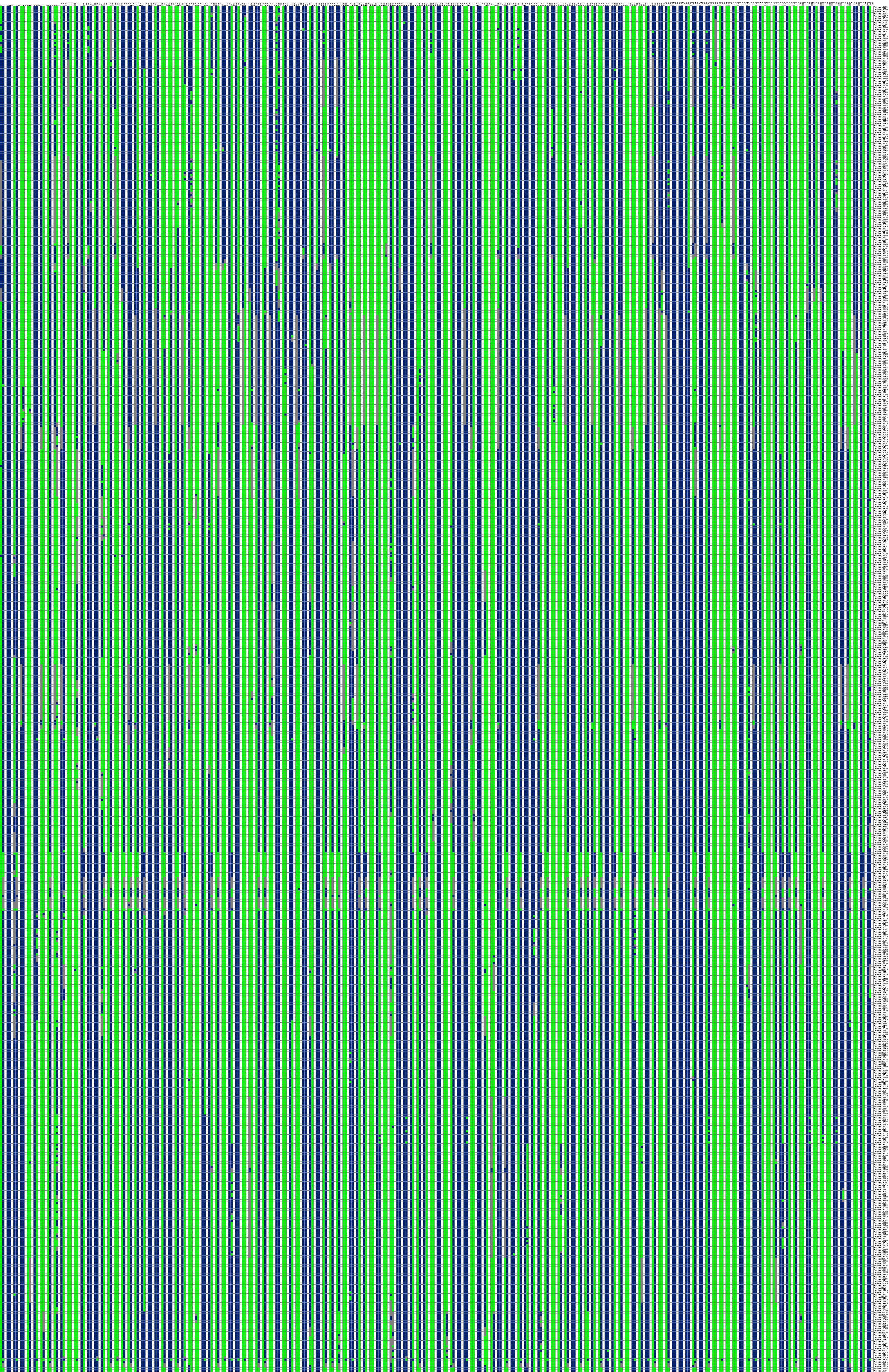

Supplement: S1 File — Each two columns represent the genotype of an individual. The first column of each individual represents ‘Beibinghong’ (the male parent); the second column of each individual represents ‘Chardonnay’ (the female parent). Rows correspond to genetic markers. Green indicates the first allele from the parent, blue refers to the second allele from the parent, and gray denotes missing data. (ZIP) [file pone.0181728.s002.zip › S1_File/chr12.sexAver.repair.tq.png]

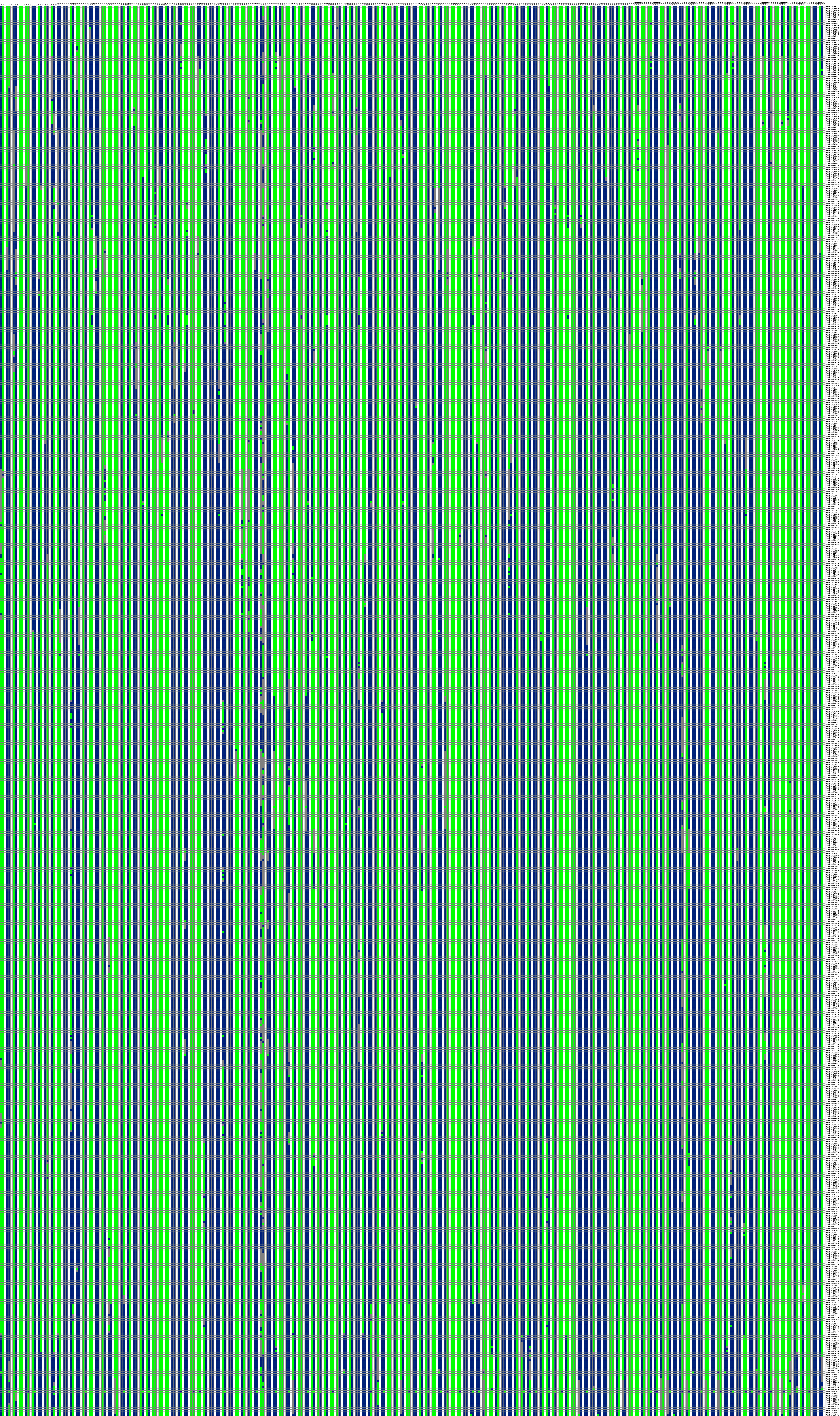

Supplement: S1 File — Each two columns represent the genotype of an individual. The first column of each individual represents ‘Beibinghong’ (the male parent); the second column of each individual represents ‘Chardonnay’ (the female parent). Rows correspond to genetic markers. Green indicates the first allele from the parent, blue refers to the second allele from the parent, and gray denotes missing data. (ZIP) [file pone.0181728.s002.zip › S1_File/chr13.sexAver.repair.tq.png]

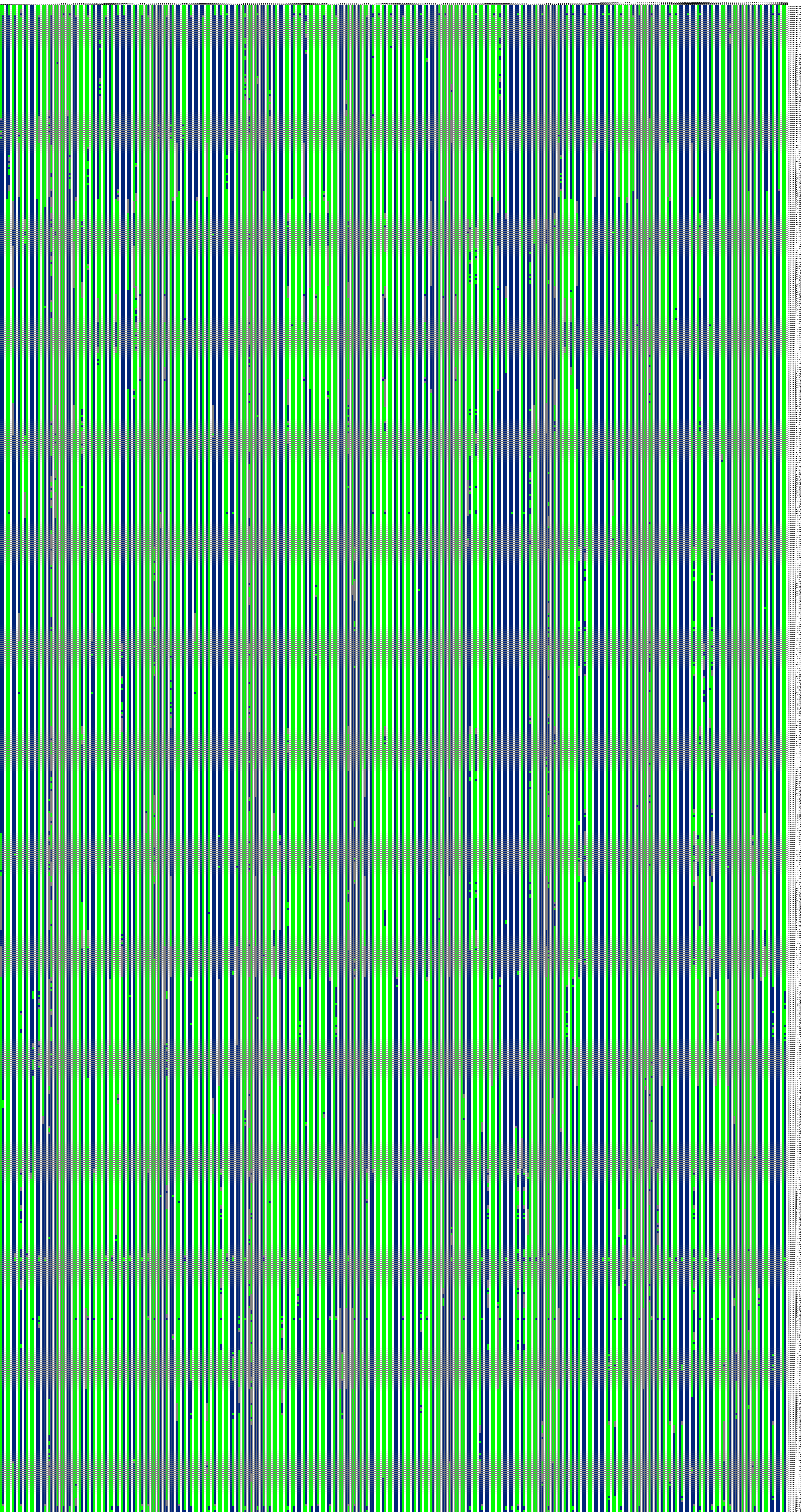

Supplement: S1 File — Each two columns represent the genotype of an individual. The first column of each individual represents ‘Beibinghong’ (the male parent); the second column of each individual represents ‘Chardonnay’ (the female parent). Rows correspond to genetic markers. Green indicates the first allele from the parent, blue refers to the second allele from the parent, and gray denotes missing data. (ZIP) [file pone.0181728.s002.zip › S1_File/chr14.sexAver.repair.tq.png]

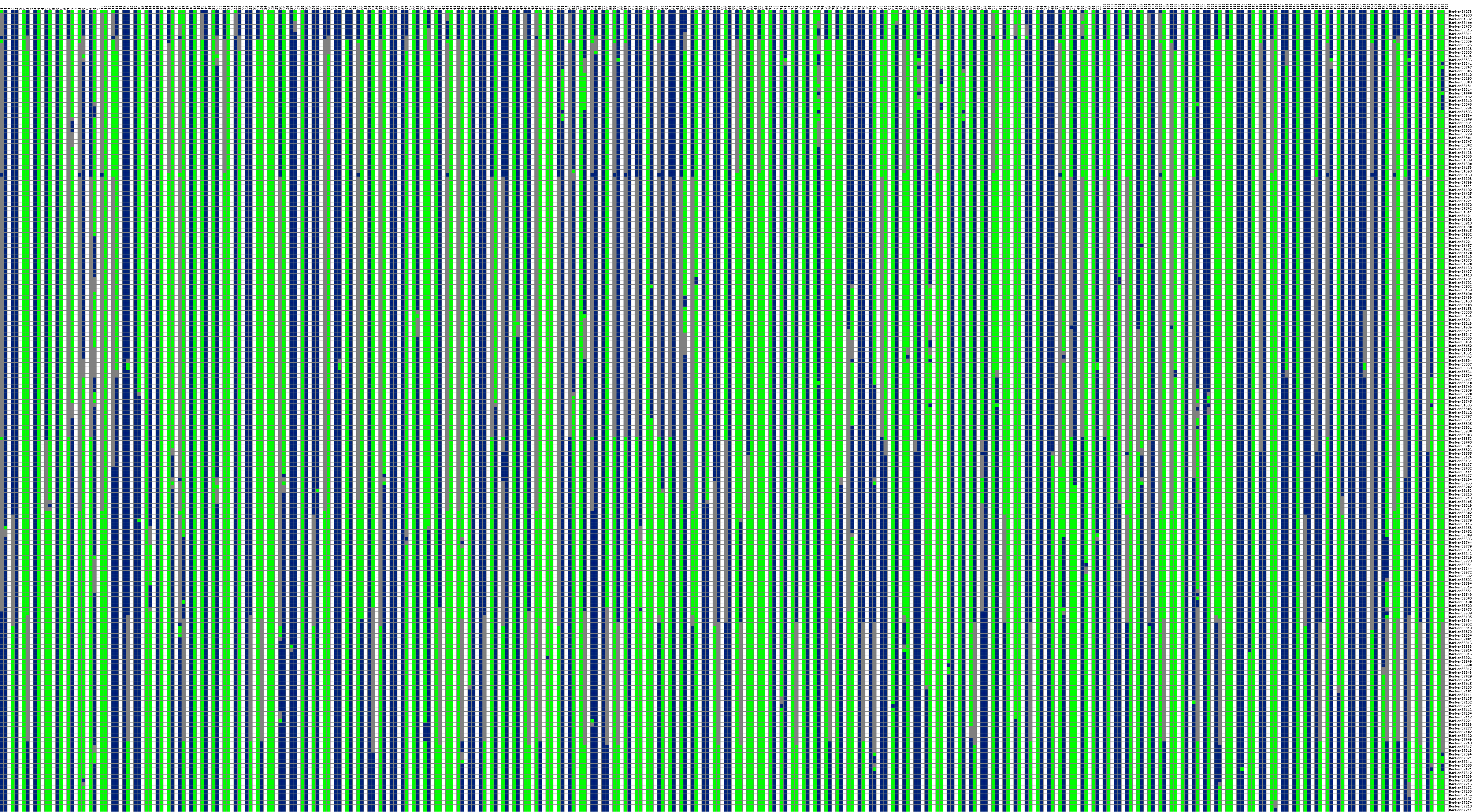

Supplement: S1 File — Each two columns represent the genotype of an individual. The first column of each individual represents ‘Beibinghong’ (the male parent); the second column of each individual represents ‘Chardonnay’ (the female parent). Rows correspond to genetic markers. Green indicates the first allele from the parent, blue refers to the second allele from the parent, and gray denotes missing data. (ZIP) [file pone.0181728.s002.zip › S1_File/chr15.sexAver.repair.tq.png]

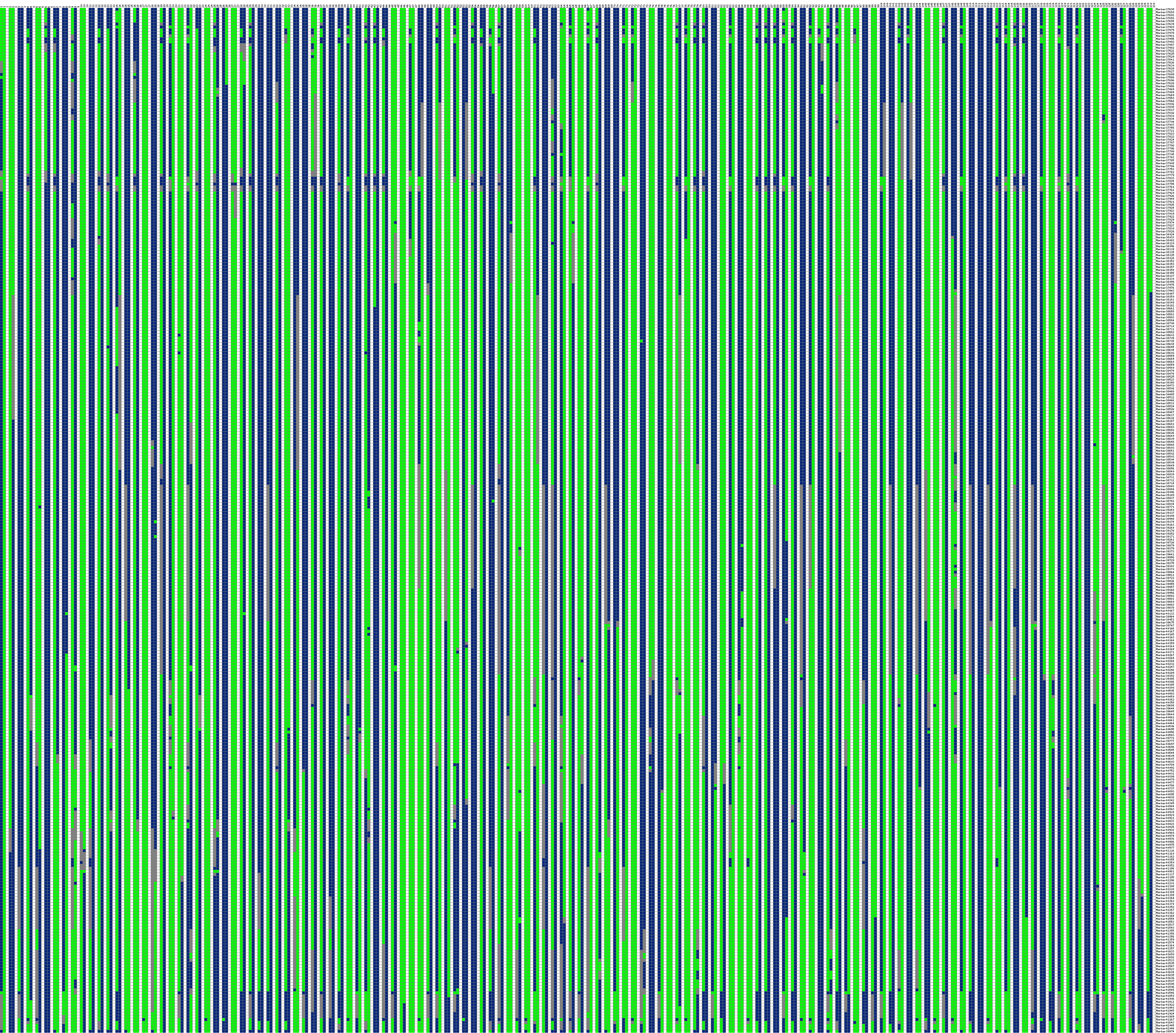

Supplement: S1 File — Each two columns represent the genotype of an individual. The first column of each individual represents ‘Beibinghong’ (the male parent); the second column of each individual represents ‘Chardonnay’ (the female parent). Rows correspond to genetic markers. Green indicates the first allele from the parent, blue refers to the second allele from the parent, and gray denotes missing data. (ZIP) [file pone.0181728.s002.zip › S1_File/chr16.sexAver.repair.tq.png]

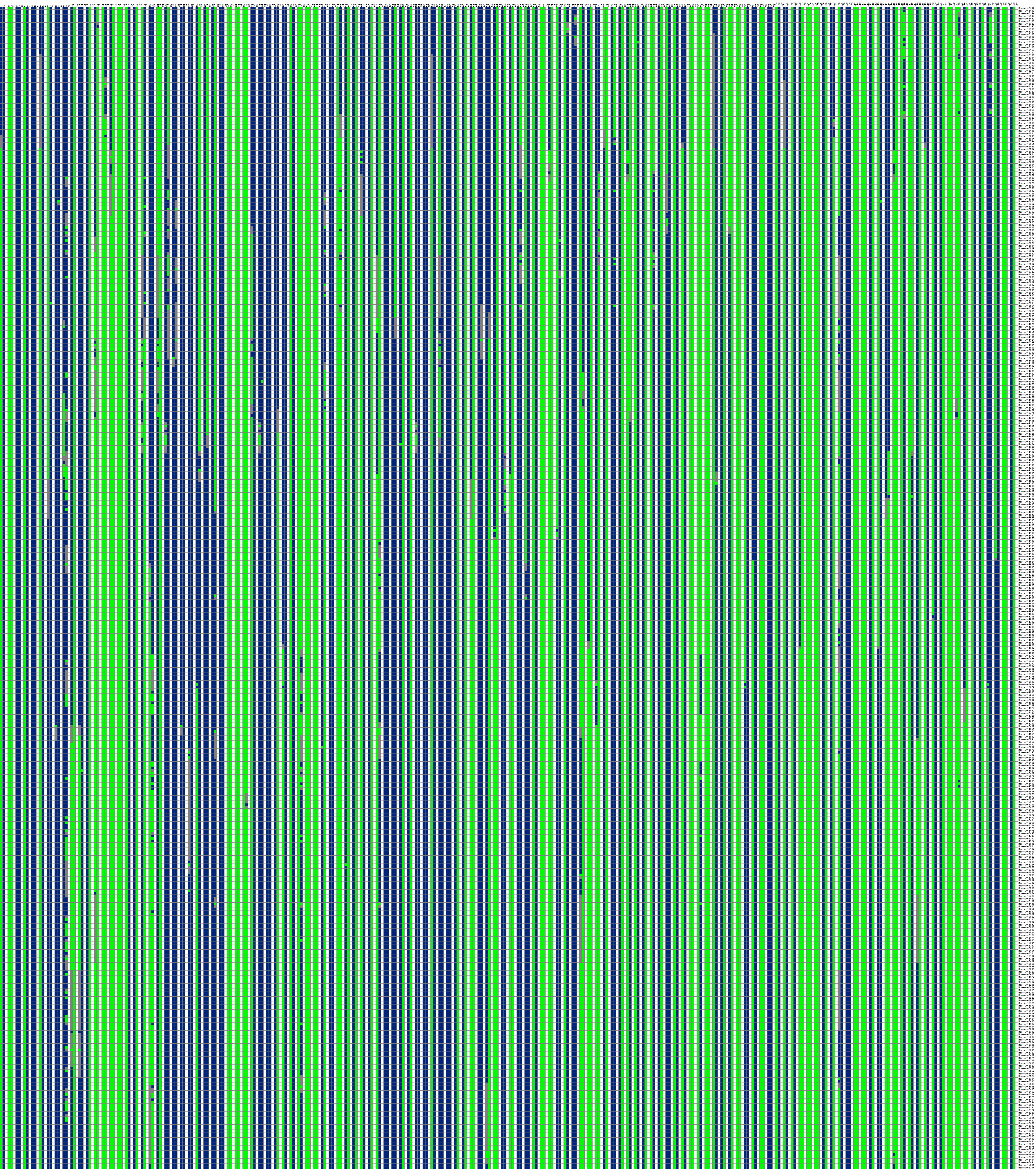

Supplement: S1 File — Each two columns represent the genotype of an individual. The first column of each individual represents ‘Beibinghong’ (the male parent); the second column of each individual represents ‘Chardonnay’ (the female parent). Rows correspond to genetic markers. Green indicates the first allele from the parent, blue refers to the second allele from the parent, and gray denotes missing data. (ZIP) [file pone.0181728.s002.zip › S1_File/chr17.sexAver.repair.tq.png]

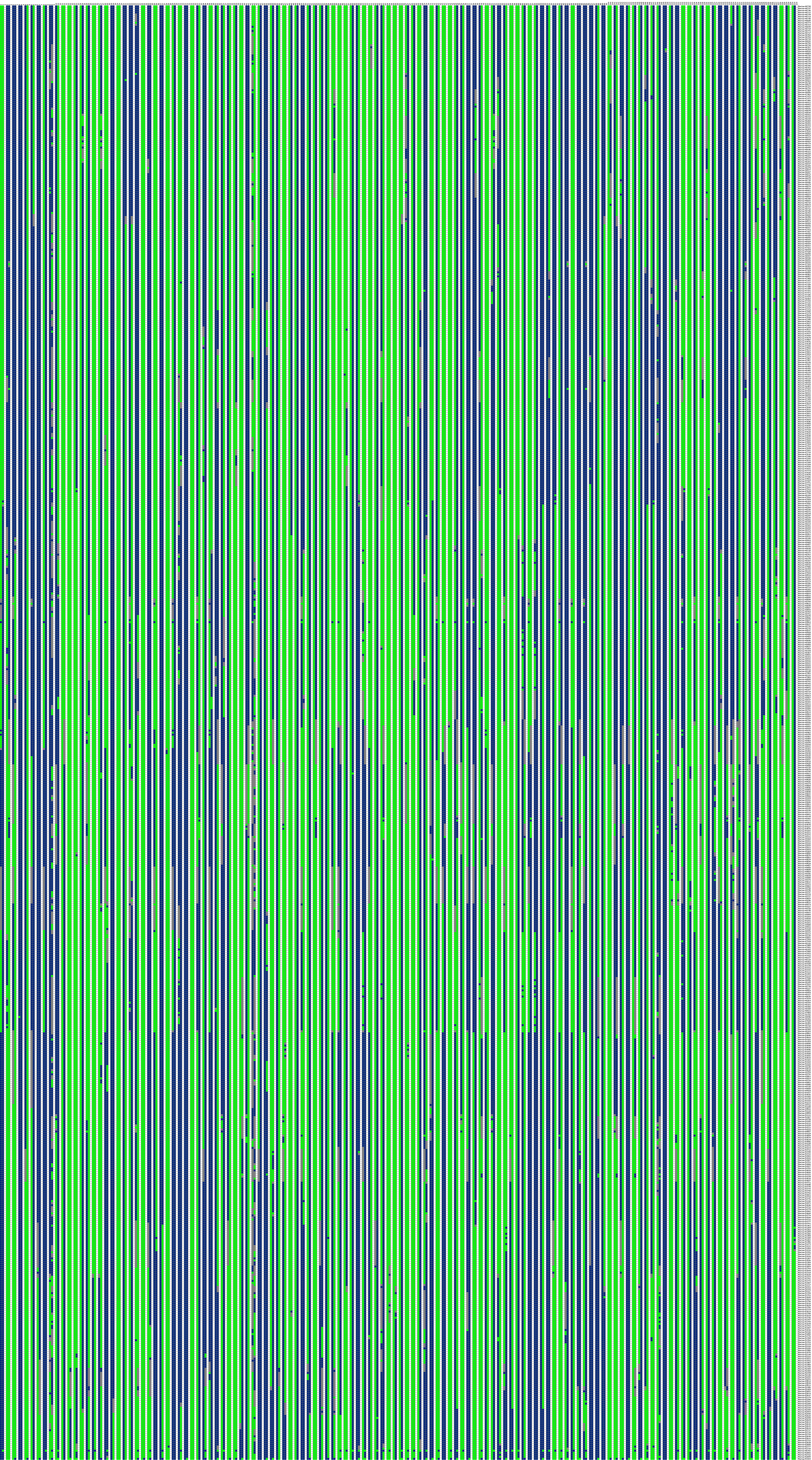

Supplement: S1 File — Each two columns represent the genotype of an individual. The first column of each individual represents ‘Beibinghong’ (the male parent); the second column of each individual represents ‘Chardonnay’ (the female parent). Rows correspond to genetic markers. Green indicates the first allele from the parent, blue refers to the second allele from the parent, and gray denotes missing data. (ZIP) [file pone.0181728.s002.zip › S1_File/chr18.sexAver.repair.tq.png]

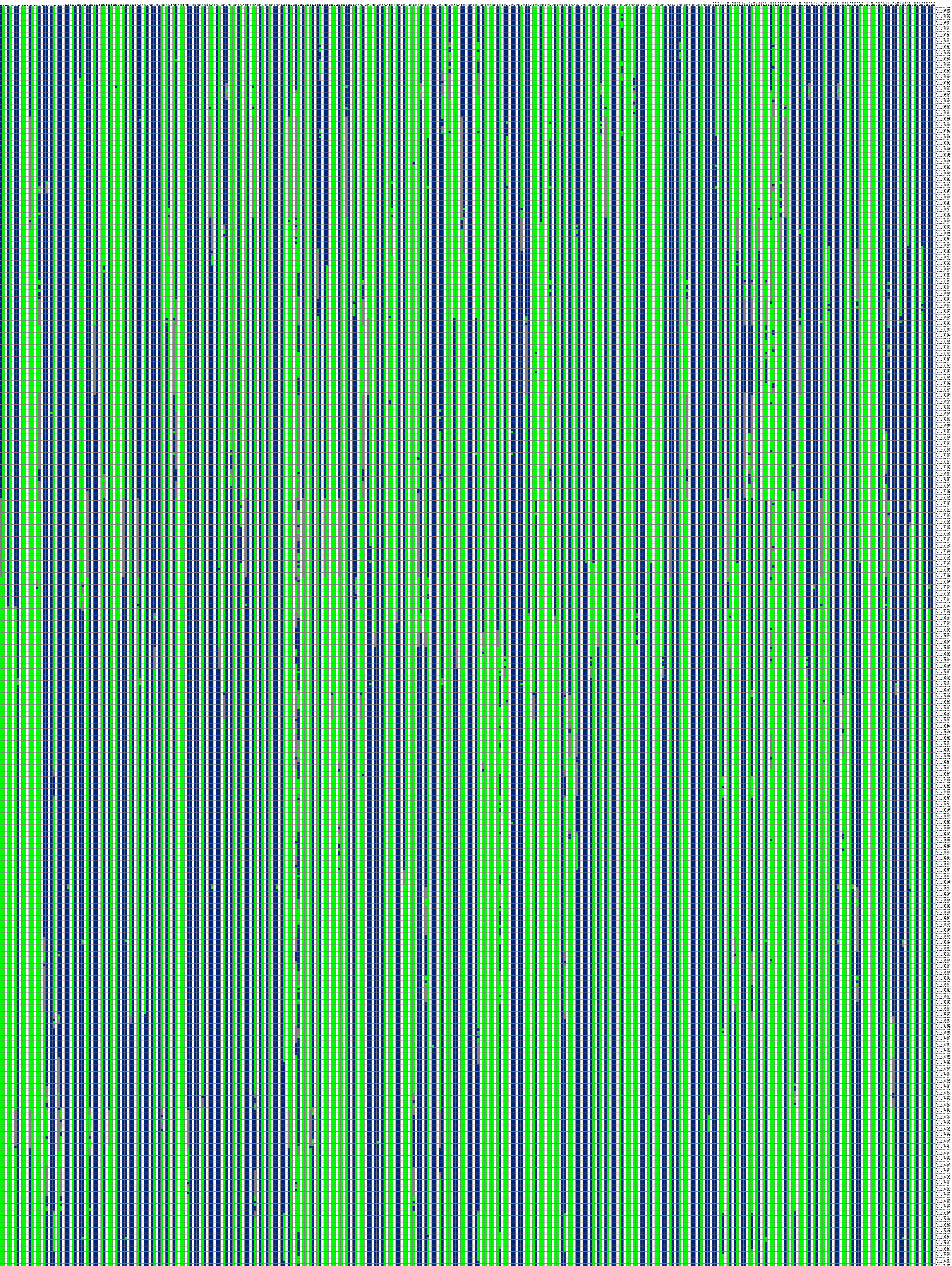

Supplement: S1 File — Each two columns represent the genotype of an individual. The first column of each individual represents ‘Beibinghong’ (the male parent); the second column of each individual represents ‘Chardonnay’ (the female parent). Rows correspond to genetic markers. Green indicates the first allele from the parent, blue refers to the second allele from the parent, and gray denotes missing data. (ZIP) [file pone.0181728.s002.zip › S1_File/chr19.sexAver.repair.tq.png]

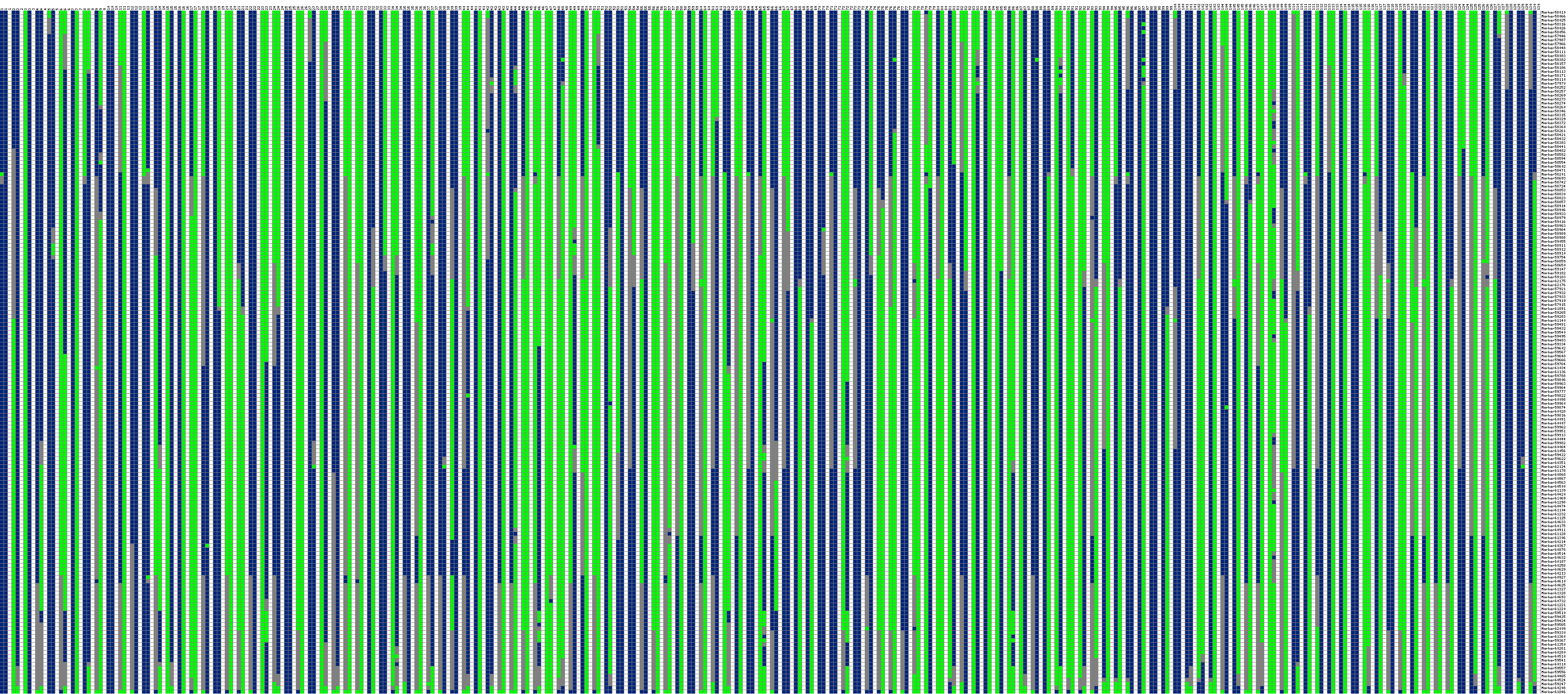

Supplement: S1 File — Each two columns represent the genotype of an individual. The first column of each individual represents ‘Beibinghong’ (the male parent); the second column of each individual represents ‘Chardonnay’ (the female parent). Rows correspond to genetic markers. Green indicates the first allele from the parent, blue refers to the second allele from the parent, and gray denotes missing data. (ZIP) [file pone.0181728.s002.zip › S1_File/chr2.sexAver.repair.tq.png]

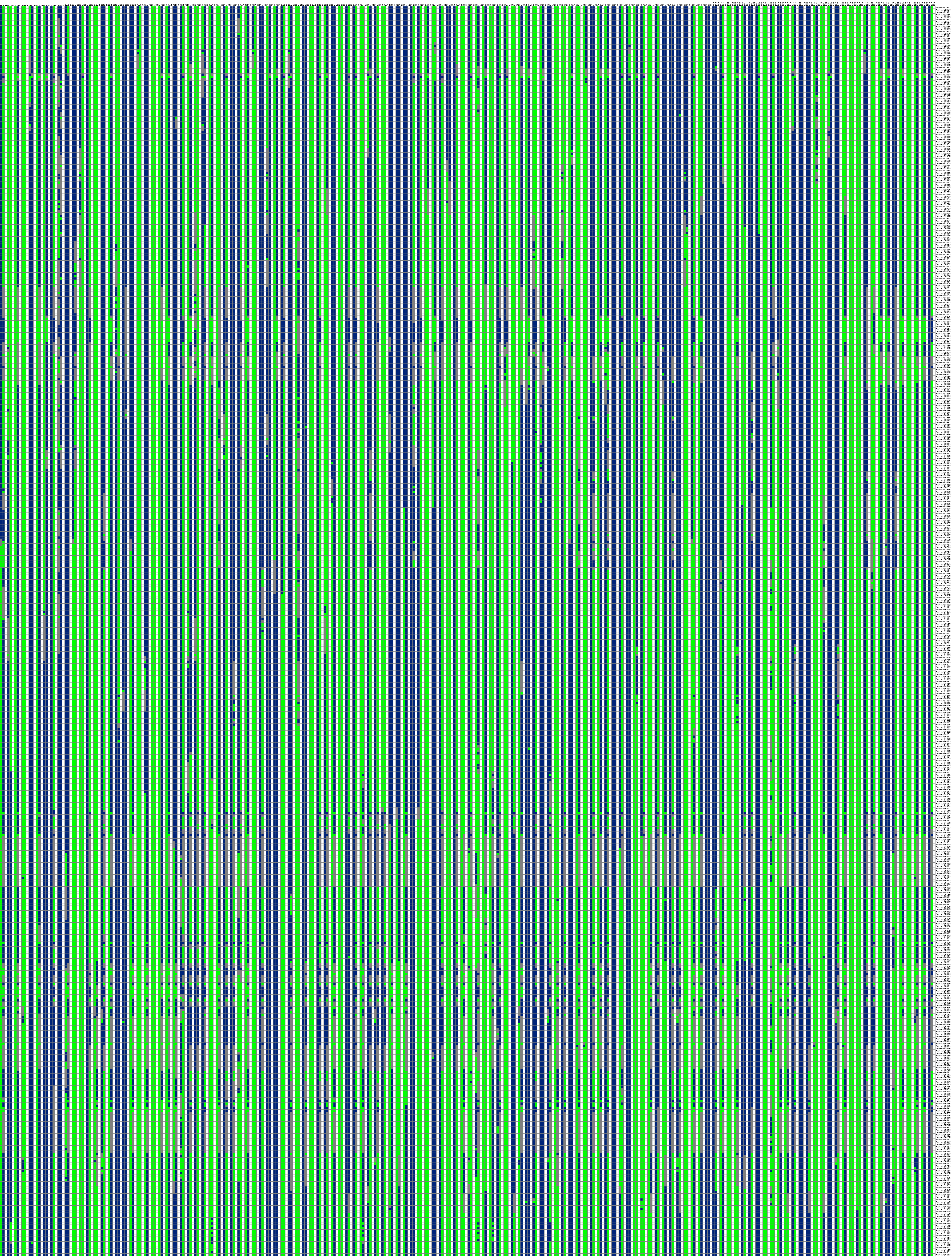

Supplement: S1 File — Each two columns represent the genotype of an individual. The first column of each individual represents ‘Beibinghong’ (the male parent); the second column of each individual represents ‘Chardonnay’ (the female parent). Rows correspond to genetic markers. Green indicates the first allele from the parent, blue refers to the second allele from the parent, and gray denotes missing data. (ZIP) [file pone.0181728.s002.zip › S1_File/chr3.sexAver.repair.tq.png]

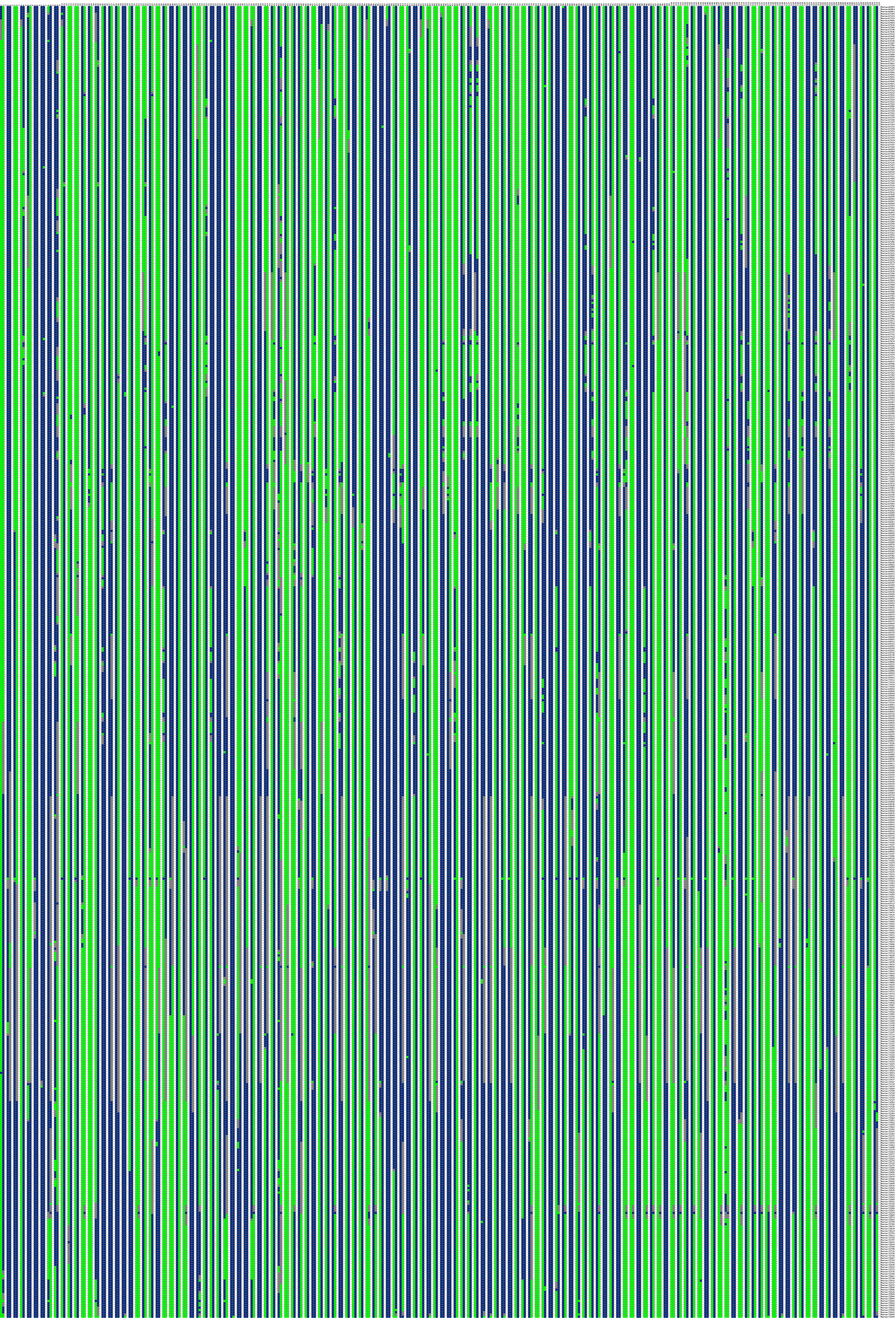

Supplement: S1 File — Each two columns represent the genotype of an individual. The first column of each individual represents ‘Beibinghong’ (the male parent); the second column of each individual represents ‘Chardonnay’ (the female parent). Rows correspond to genetic markers. Green indicates the first allele from the parent, blue refers to the second allele from the parent, and gray denotes missing data. (ZIP) [file pone.0181728.s002.zip › S1_File/chr4.sexAver.repair.tq.png]

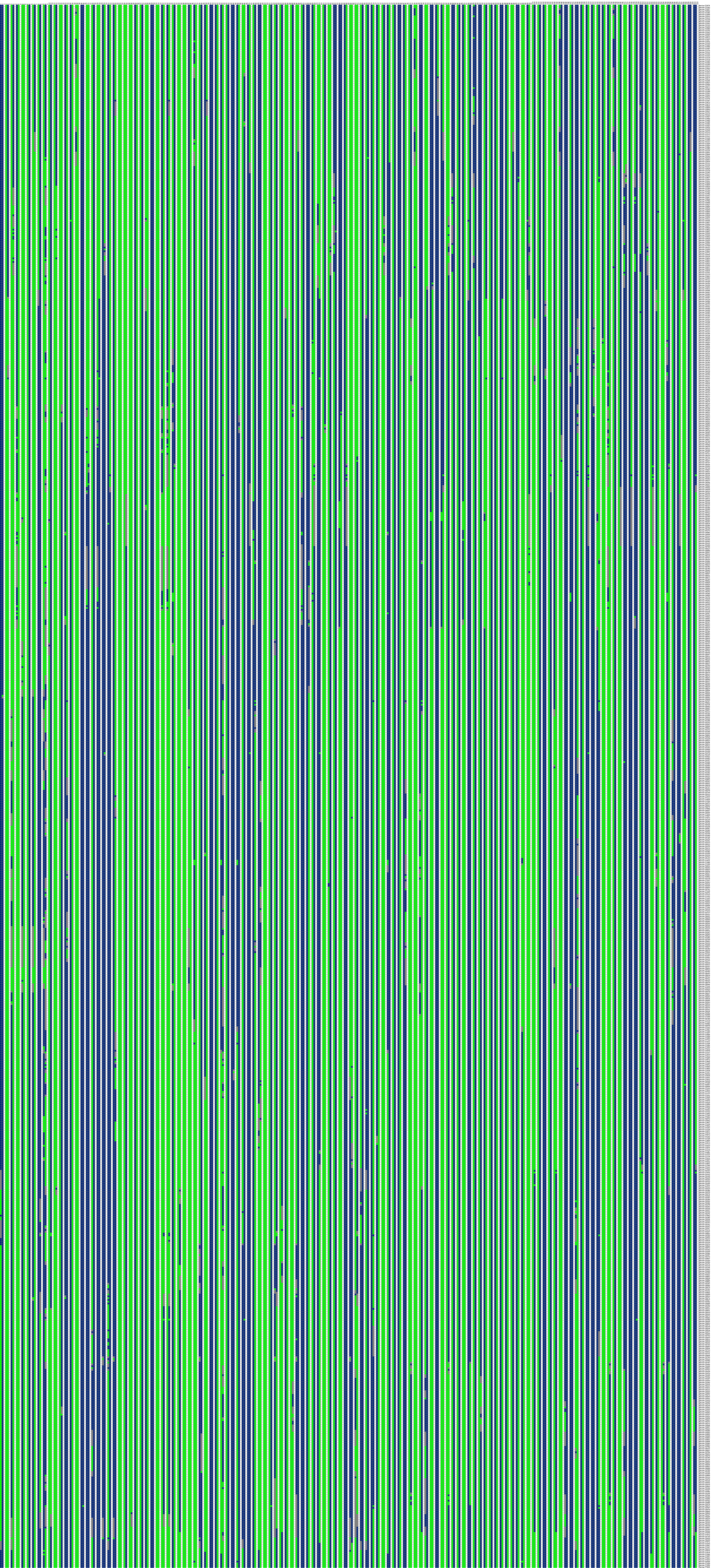

Supplement: S1 File — Each two columns represent the genotype of an individual. The first column of each individual represents ‘Beibinghong’ (the male parent); the second column of each individual represents ‘Chardonnay’ (the female parent). Rows correspond to genetic markers. Green indicates the first allele from the parent, blue refers to the second allele from the parent, and gray denotes missing data. (ZIP) [file pone.0181728.s002.zip › S1_File/chr5.sexAver.repair.tq.png]

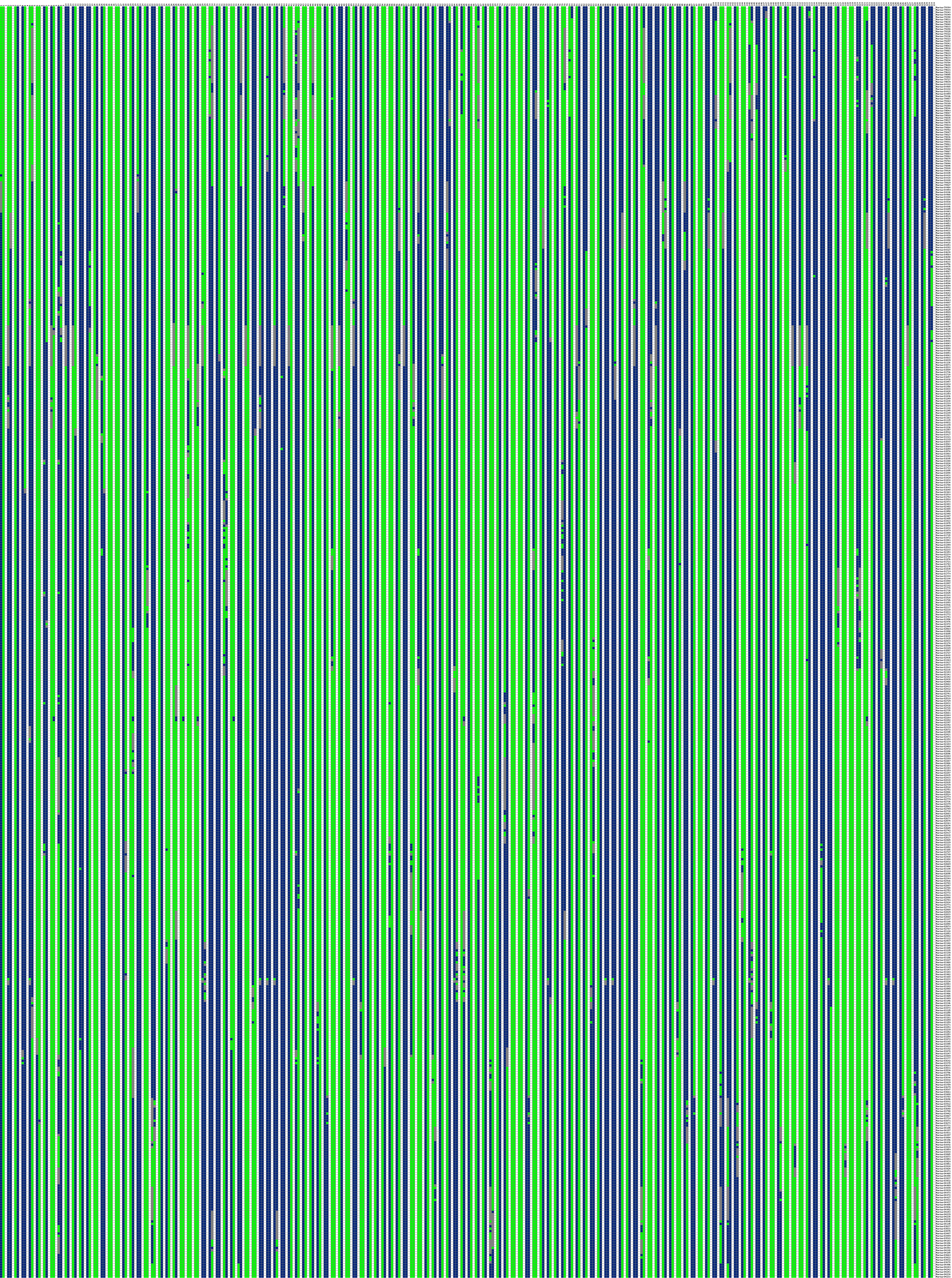

Supplement: S1 File — Each two columns represent the genotype of an individual. The first column of each individual represents ‘Beibinghong’ (the male parent); the second column of each individual represents ‘Chardonnay’ (the female parent). Rows correspond to genetic markers. Green indicates the first allele from the parent, blue refers to the second allele from the parent, and gray denotes missing data. (ZIP) [file pone.0181728.s002.zip › S1_File/chr6.sexAver.repair.tq.png]

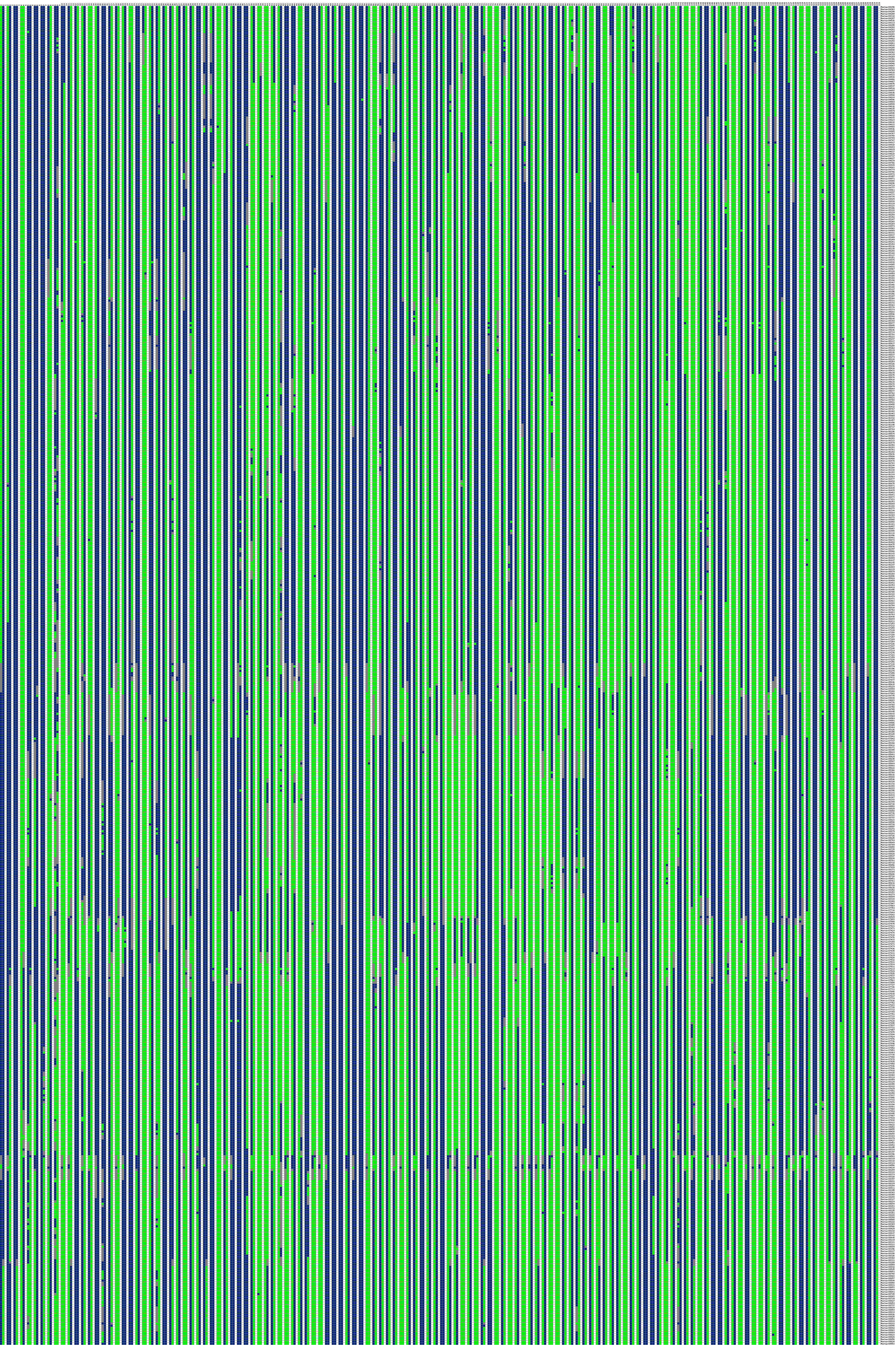

Supplement: S1 File — Each two columns represent the genotype of an individual. The first column of each individual represents ‘Beibinghong’ (the male parent); the second column of each individual represents ‘Chardonnay’ (the female parent). Rows correspond to genetic markers. Green indicates the first allele from the parent, blue refers to the second allele from the parent, and gray denotes missing data. (ZIP) [file pone.0181728.s002.zip › S1_File/chr7.sexAver.repair.tq.png]

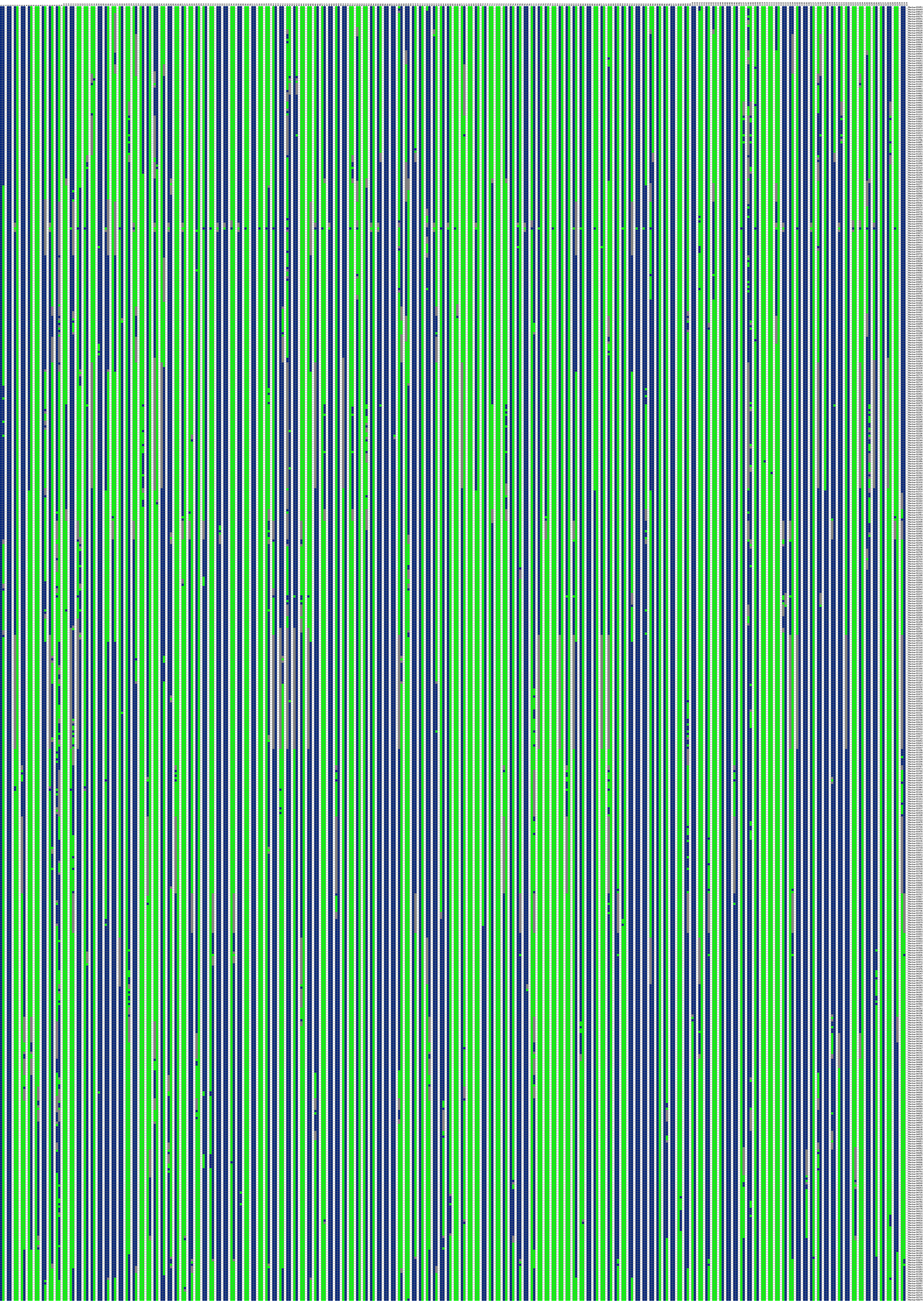

Supplement: S1 File — Each two columns represent the genotype of an individual. The first column of each individual represents ‘Beibinghong’ (the male parent); the second column of each individual represents ‘Chardonnay’ (the female parent). Rows correspond to genetic markers. Green indicates the first allele from the parent, blue refers to the second allele from the parent, and gray denotes missing data. (ZIP) [file pone.0181728.s002.zip › S1_File/chr8.sexAver.repair.tq.png]

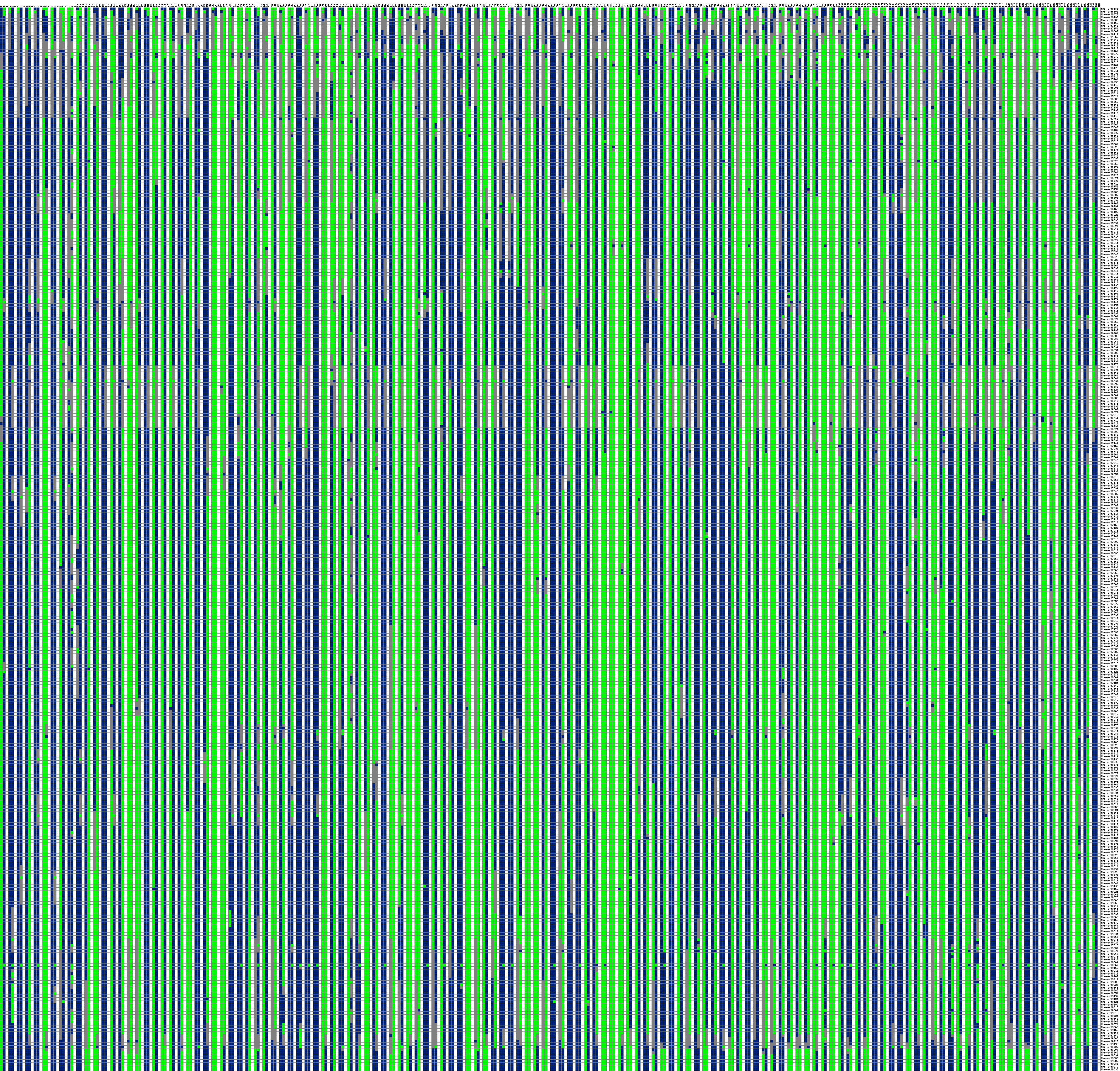

Supplement: S1 File — Each two columns represent the genotype of an individual. The first column of each individual represents ‘Beibinghong’ (the male parent); the second column of each individual represents ‘Chardonnay’ (the female parent). Rows correspond to genetic markers. Green indicates the first allele from the parent, blue refers to the second allele from the parent, and gray denotes missing data. (ZIP) [file pone.0181728.s002.zip › S1_File/chr9.sexAver.repair.tq.png]

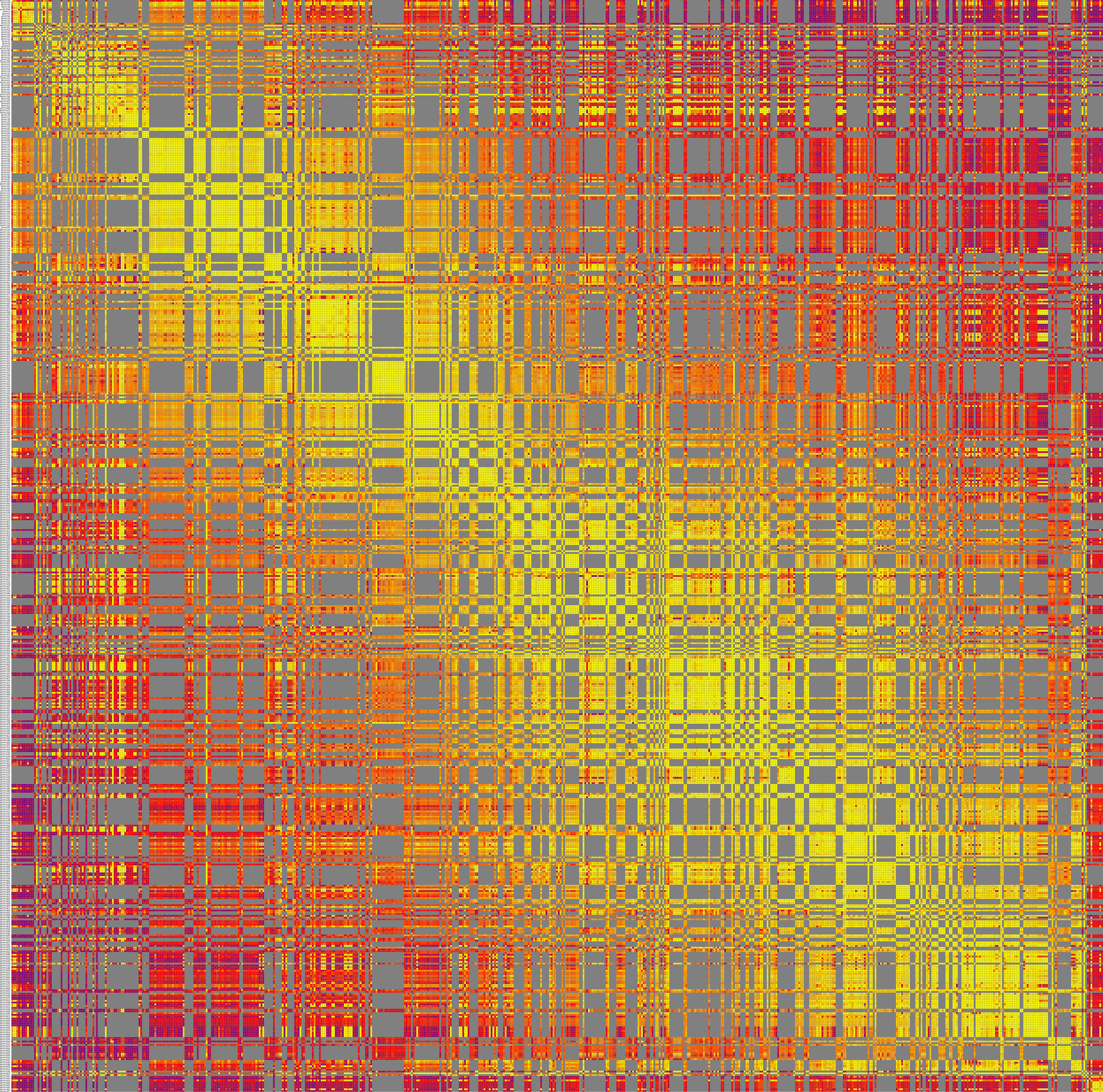

Supplement: S2 File — Each cell represents the recombination rate of two markers. Yellow and purple indicate lower and higher recombination rates, respectively. Gray denotes missing data. (ZIP) [file pone.0181728.s003.zip › S2_File/chr1.sexAver.r.heatMap.png]

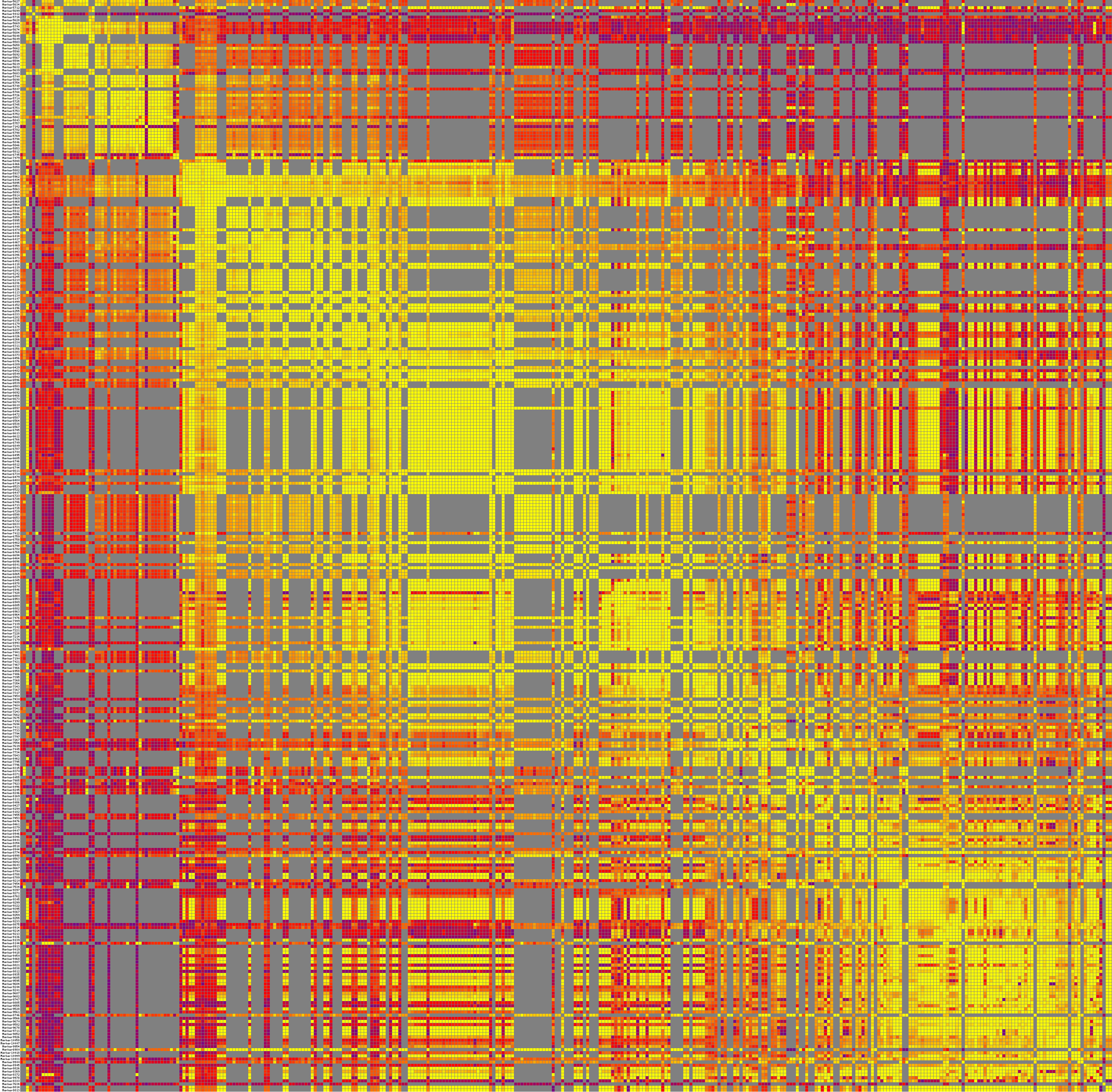

Supplement: S2 File — Each cell represents the recombination rate of two markers. Yellow and purple indicate lower and higher recombination rates, respectively. Gray denotes missing data. (ZIP) [file pone.0181728.s003.zip › S2_File/chr10.sexAver.r.heatMap.png]

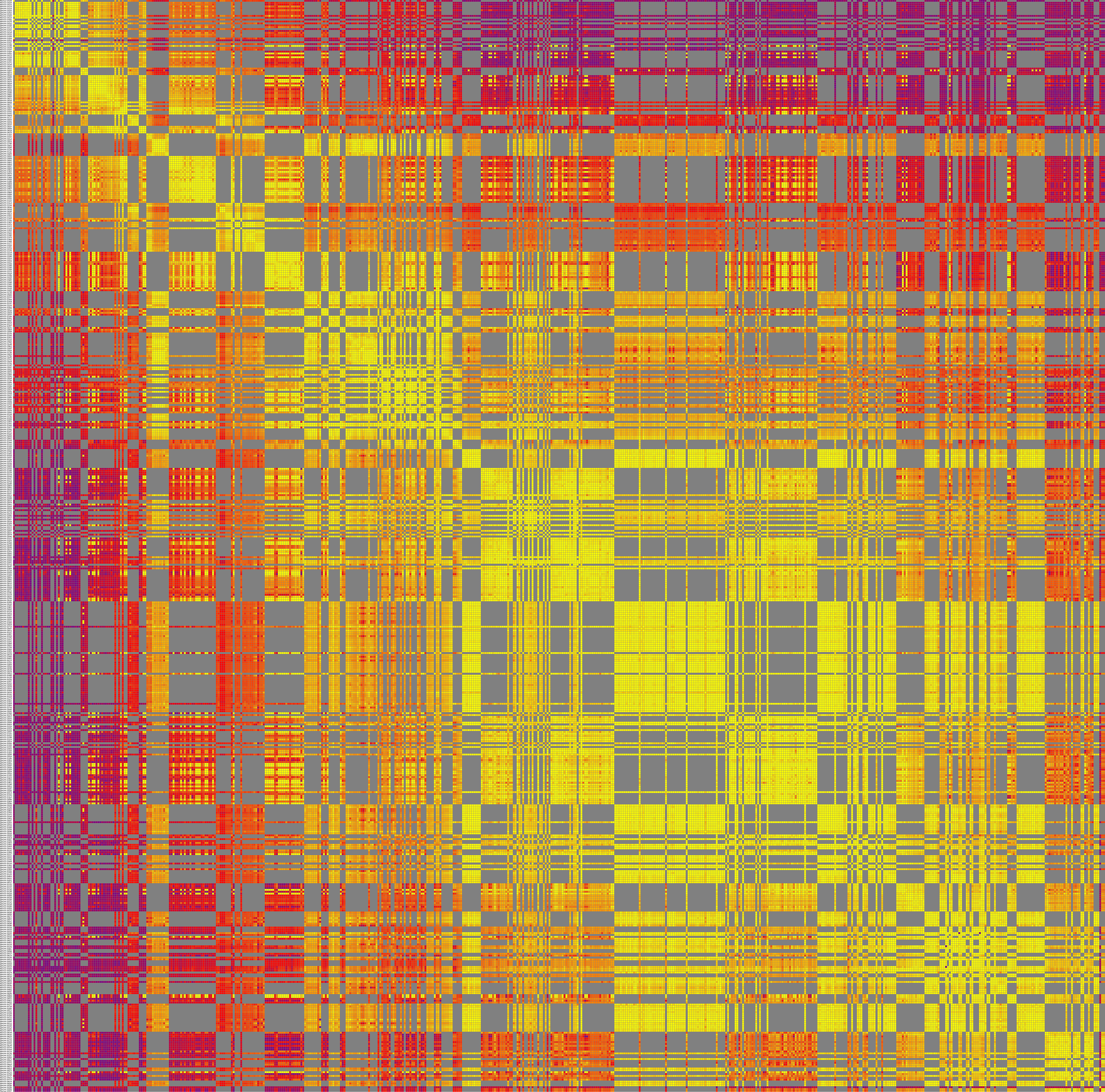

Supplement: S2 File — Each cell represents the recombination rate of two markers. Yellow and purple indicate lower and higher recombination rates, respectively. Gray denotes missing data. (ZIP) [file pone.0181728.s003.zip › S2_File/chr11.sexAver.r.heatMap.png]

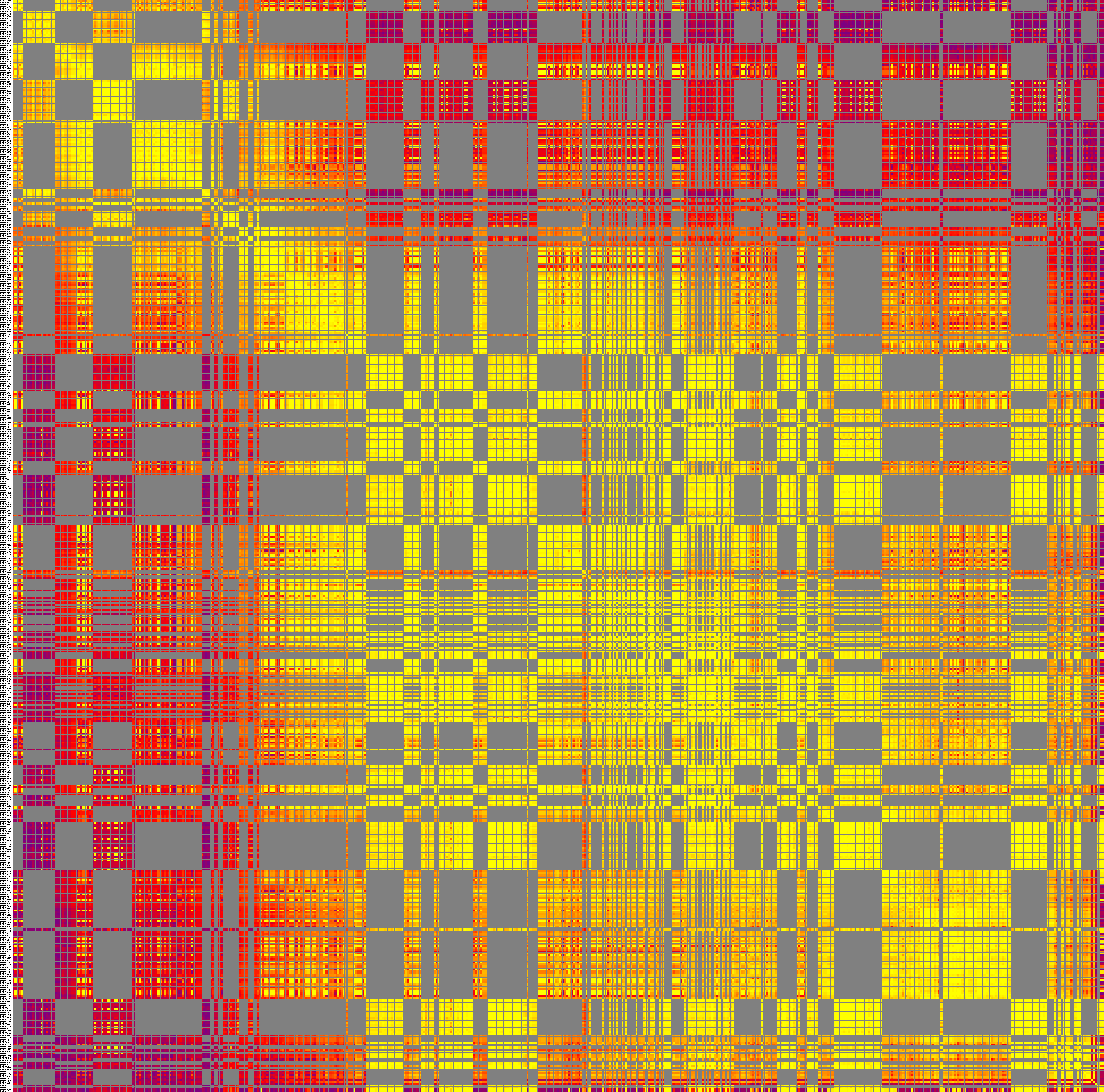

Supplement: S2 File — Each cell represents the recombination rate of two markers. Yellow and purple indicate lower and higher recombination rates, respectively. Gray denotes missing data. (ZIP) [file pone.0181728.s003.zip › S2_File/chr12.sexAver.r.heatMap.png]

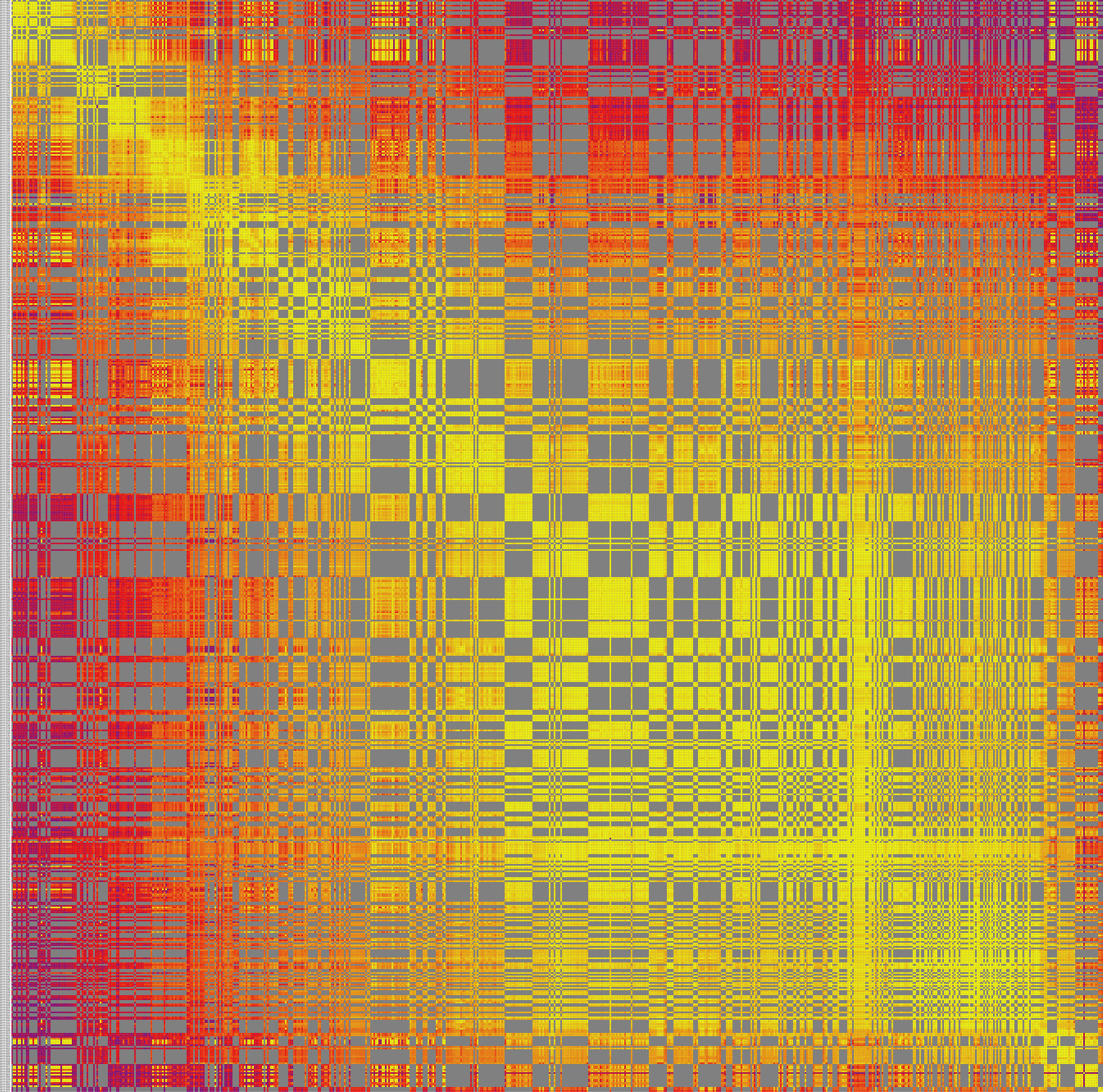

Supplement: S2 File — Each cell represents the recombination rate of two markers. Yellow and purple indicate lower and higher recombination rates, respectively. Gray denotes missing data. (ZIP) [file pone.0181728.s003.zip › S2_File/chr13.sexAver.r.heatMap.png]

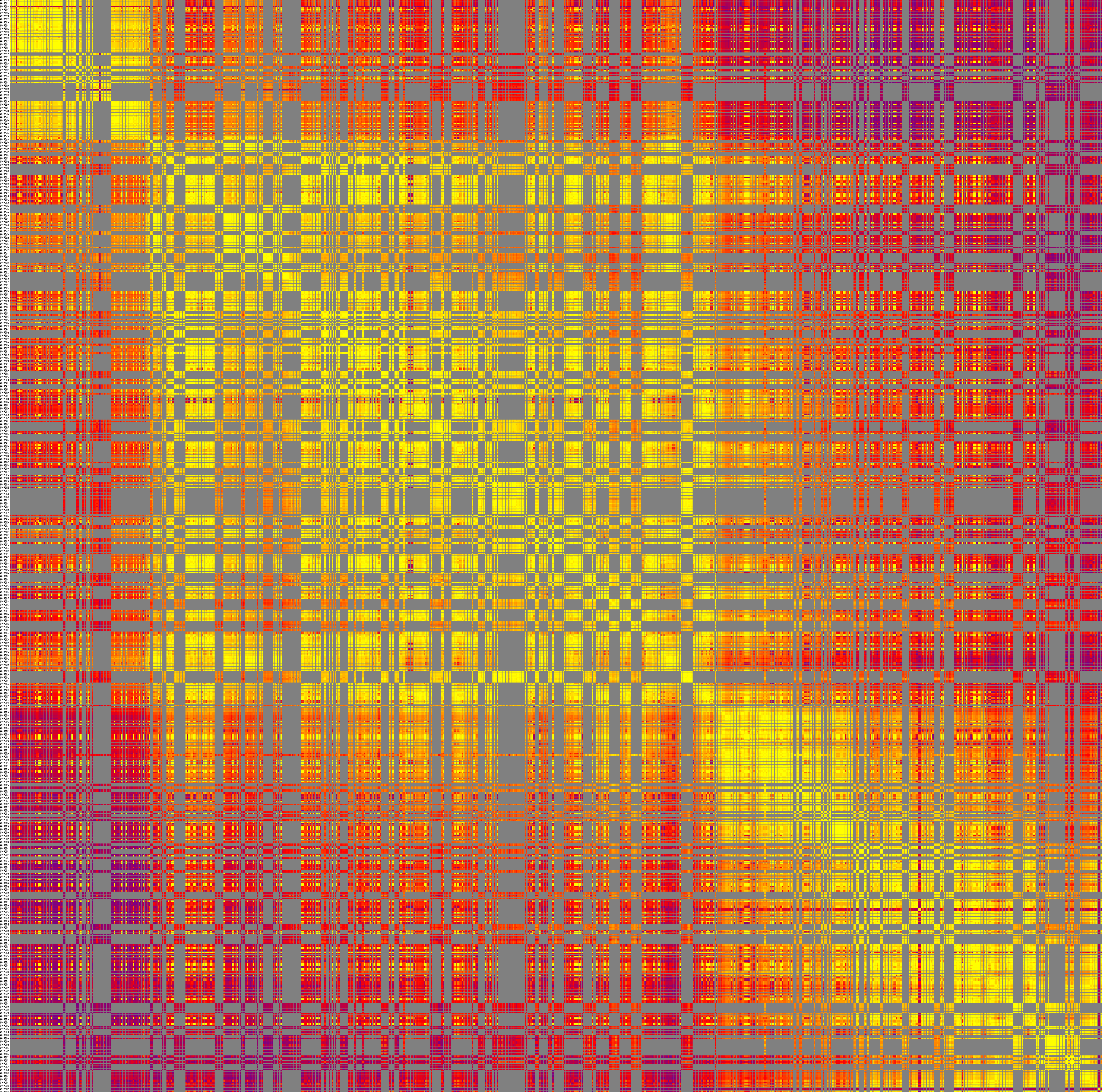

Supplement: S2 File — Each cell represents the recombination rate of two markers. Yellow and purple indicate lower and higher recombination rates, respectively. Gray denotes missing data. (ZIP) [file pone.0181728.s003.zip › S2_File/chr14.sexAver.r.heatMap.png]

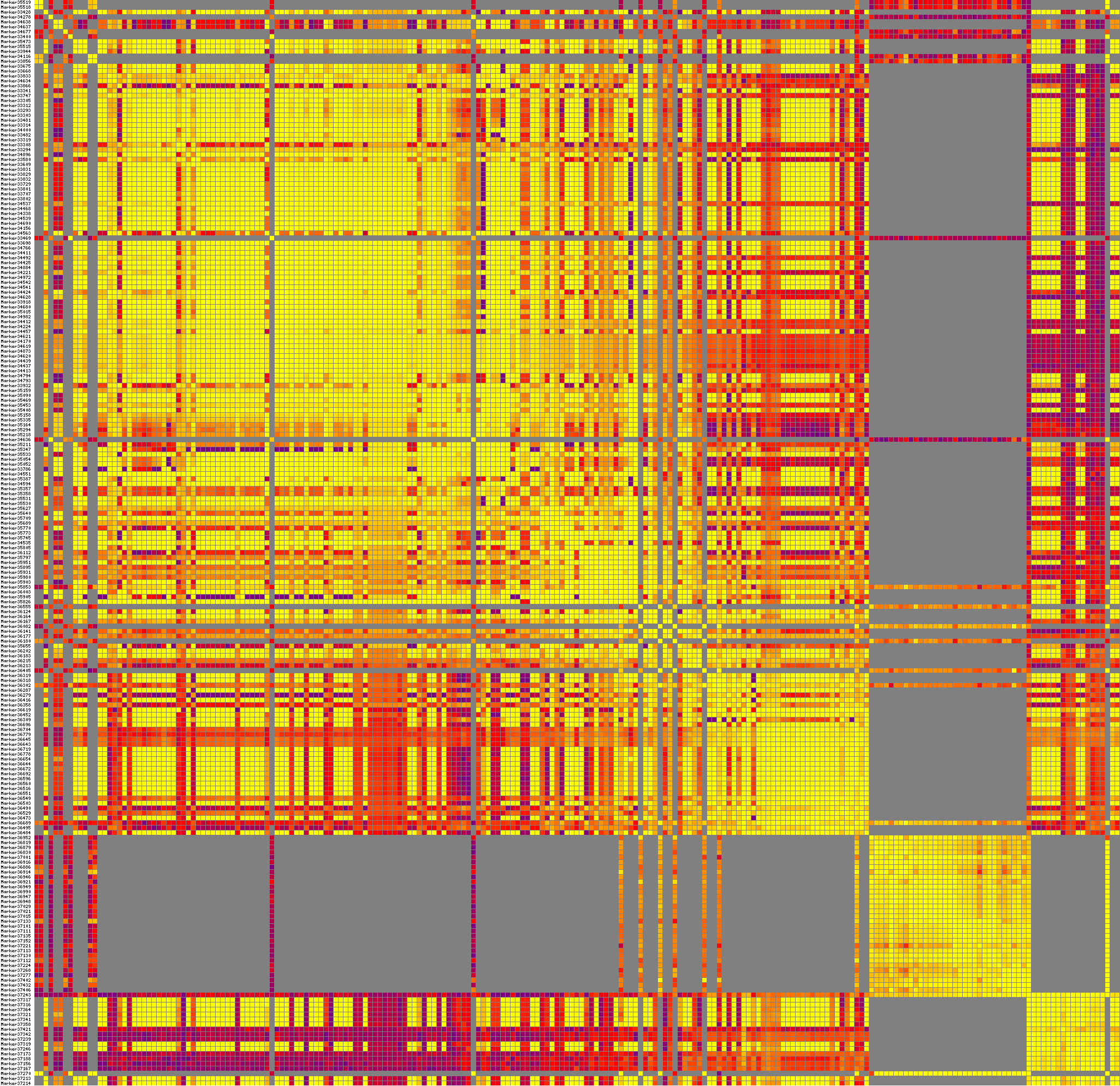

Supplement: S2 File — Each cell represents the recombination rate of two markers. Yellow and purple indicate lower and higher recombination rates, respectively. Gray denotes missing data. (ZIP) [file pone.0181728.s003.zip › S2_File/chr15.sexAver.r.heatMap.png]

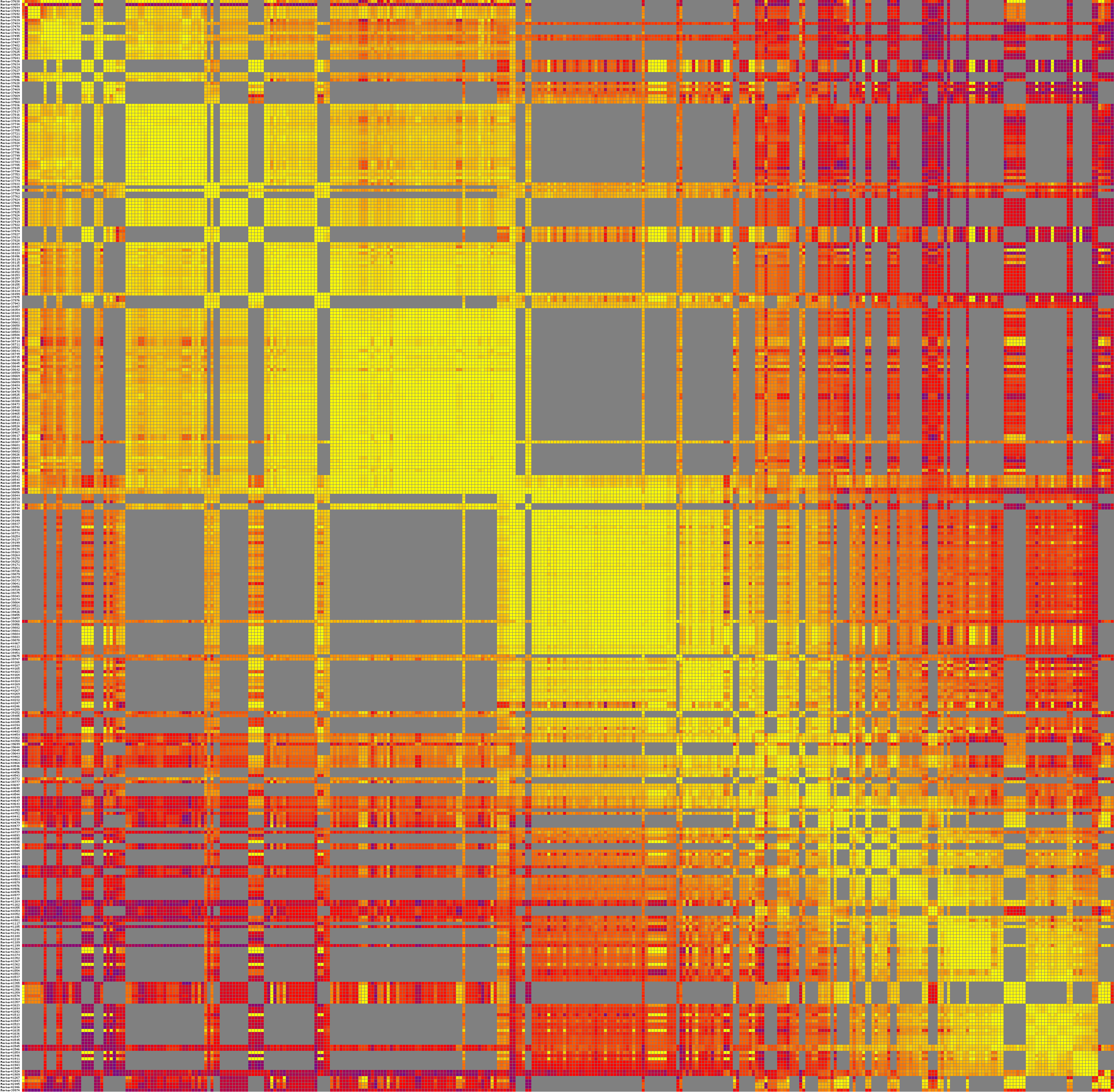

Supplement: S2 File — Each cell represents the recombination rate of two markers. Yellow and purple indicate lower and higher recombination rates, respectively. Gray denotes missing data. (ZIP) [file pone.0181728.s003.zip › S2_File/chr16.sexAver.r.heatMap.png]

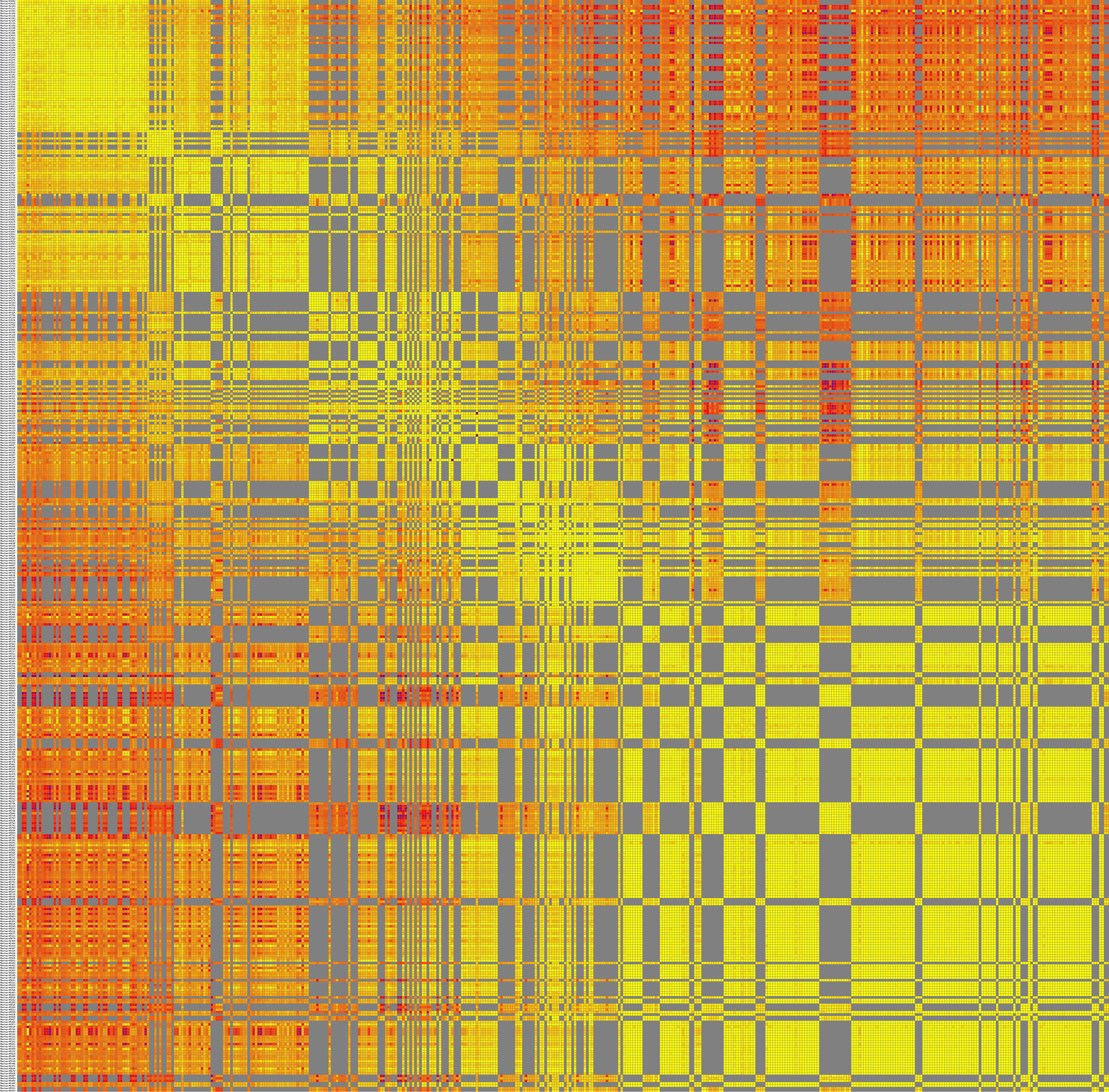

Supplement: S2 File — Each cell represents the recombination rate of two markers. Yellow and purple indicate lower and higher recombination rates, respectively. Gray denotes missing data. (ZIP) [file pone.0181728.s003.zip › S2_File/chr17.sexAver.r.heatMap.png]

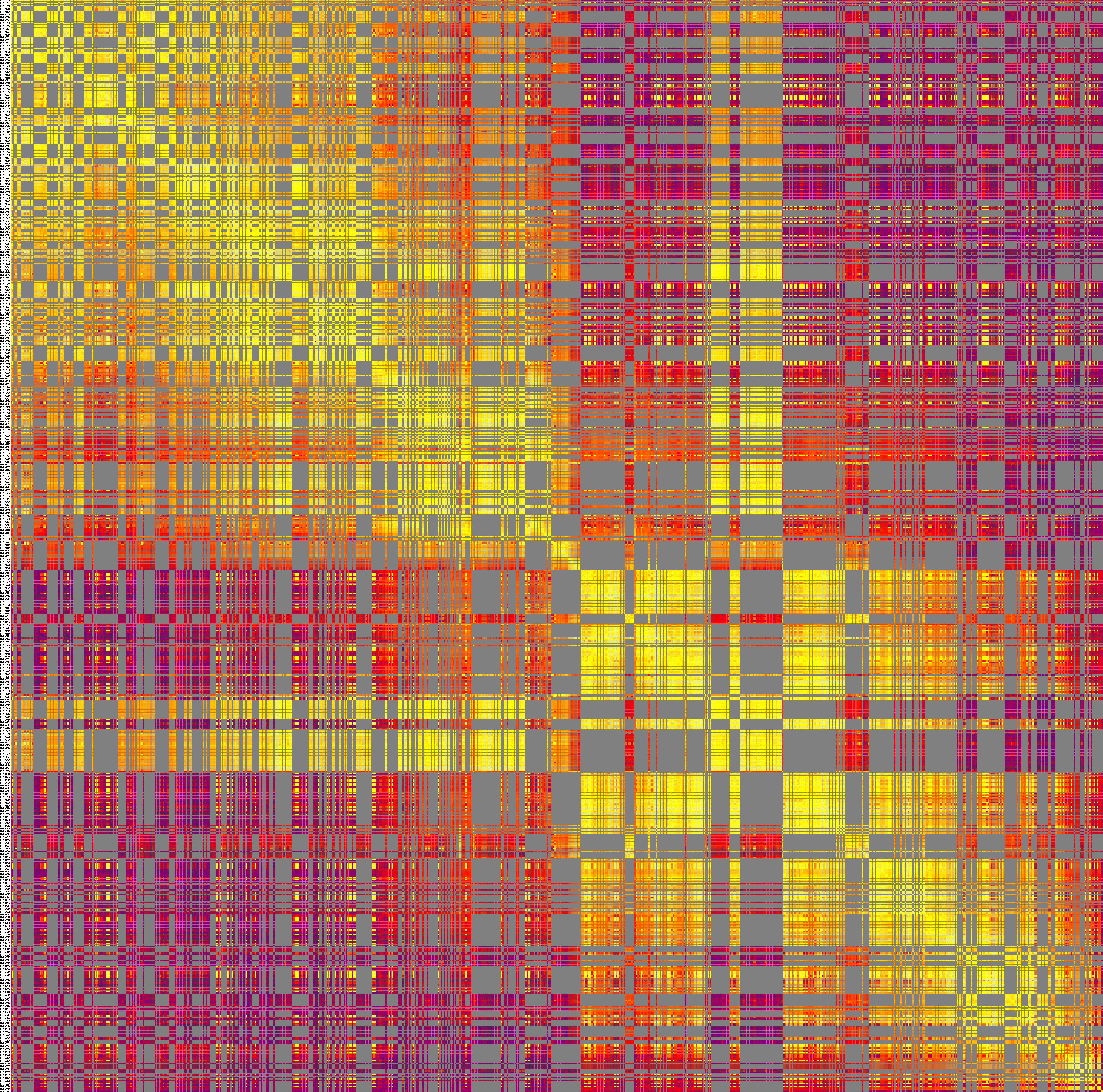

Supplement: S2 File — Each cell represents the recombination rate of two markers. Yellow and purple indicate lower and higher recombination rates, respectively. Gray denotes missing data. (ZIP) [file pone.0181728.s003.zip › S2_File/chr18.sexAver.r.heatMap.png]

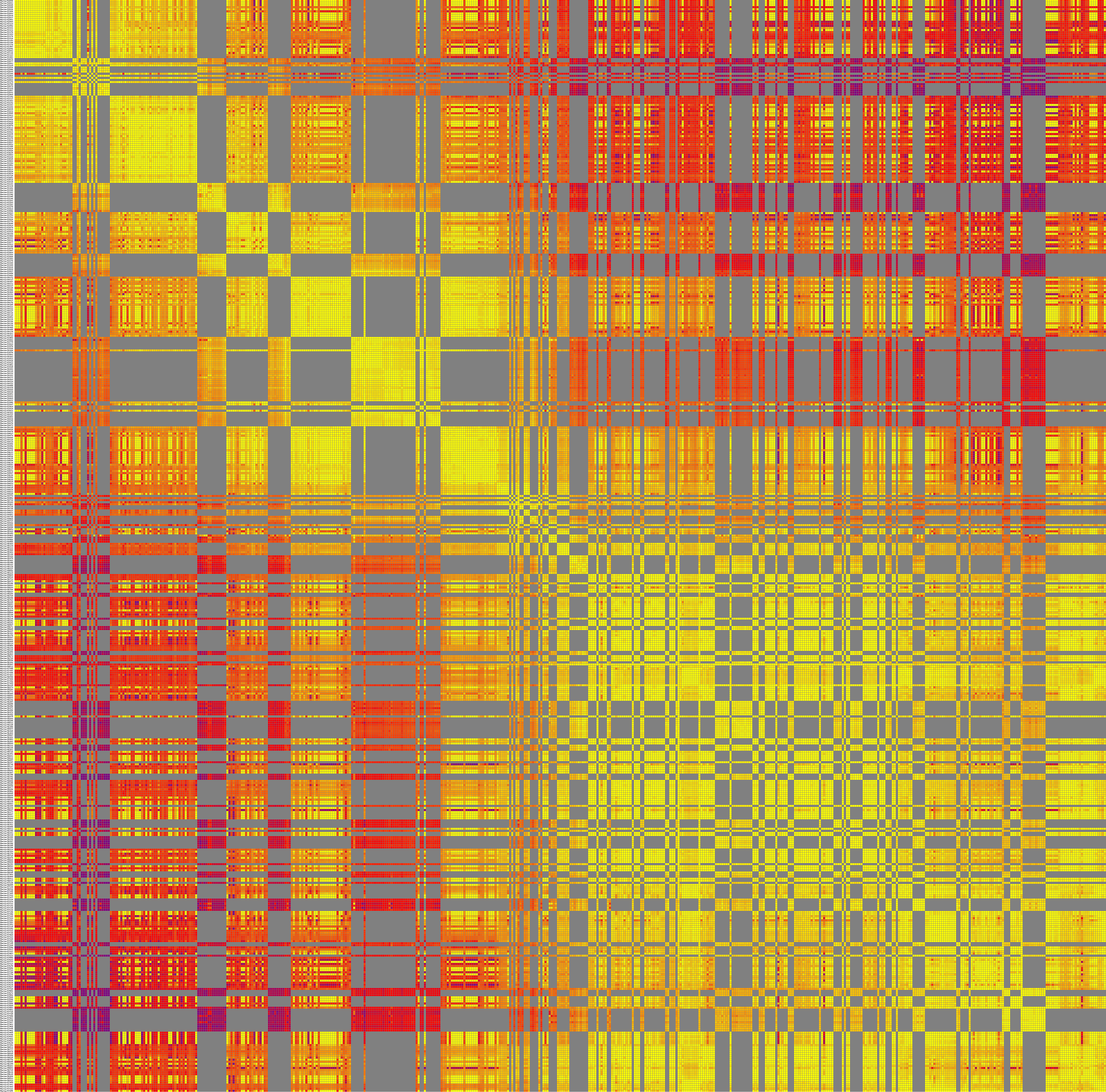

Supplement: S2 File — Each cell represents the recombination rate of two markers. Yellow and purple indicate lower and higher recombination rates, respectively. Gray denotes missing data. (ZIP) [file pone.0181728.s003.zip › S2_File/chr19.sexAver.r.heatMap.png]

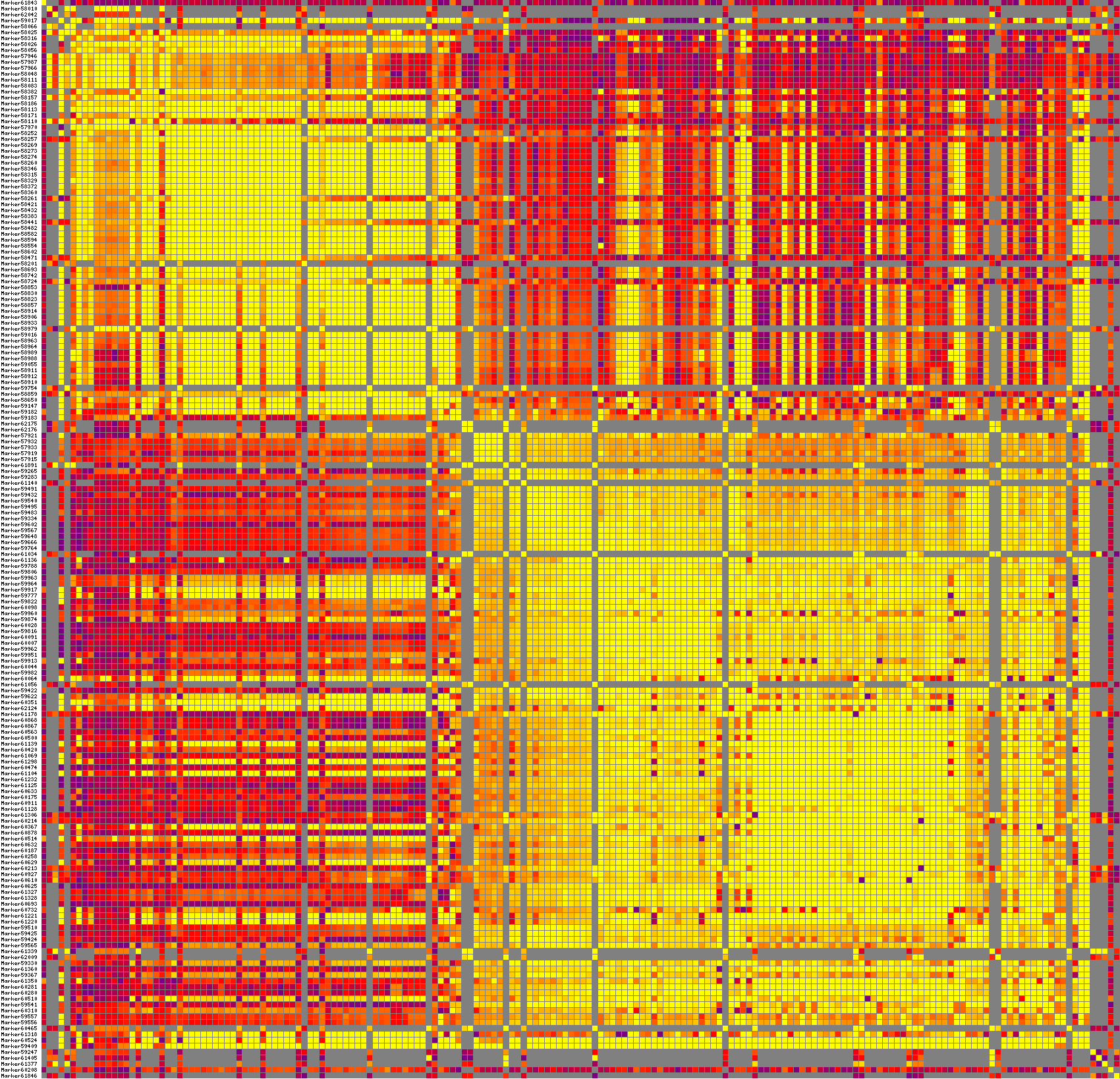

Supplement: S2 File — Each cell represents the recombination rate of two markers. Yellow and purple indicate lower and higher recombination rates, respectively. Gray denotes missing data. (ZIP) [file pone.0181728.s003.zip › S2_File/chr2.sexAver.r.heatMap.png]

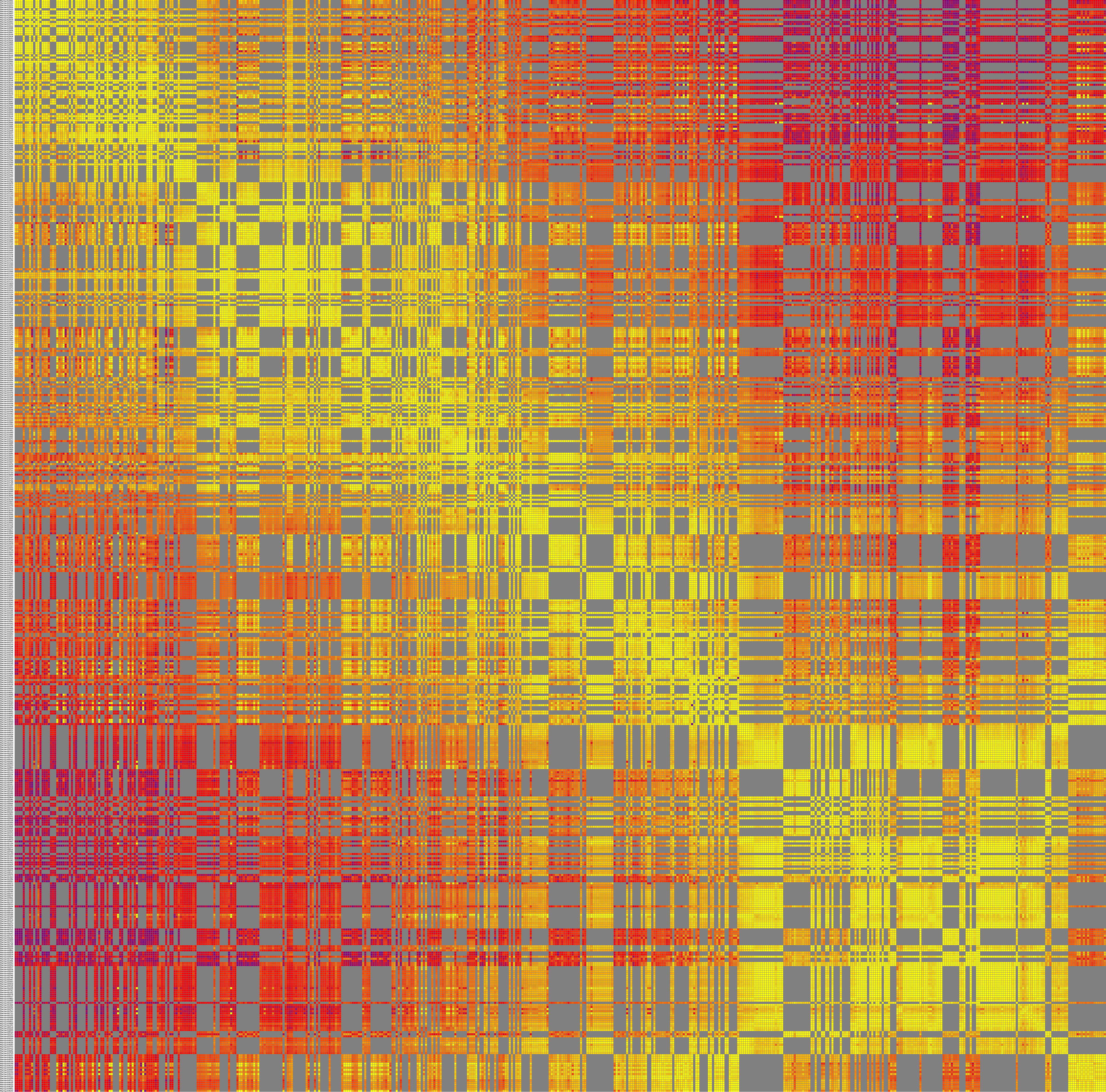

Supplement: S2 File — Each cell represents the recombination rate of two markers. Yellow and purple indicate lower and higher recombination rates, respectively. Gray denotes missing data. (ZIP) [file pone.0181728.s003.zip › S2_File/chr3.sexAver.r.heatMap.png]

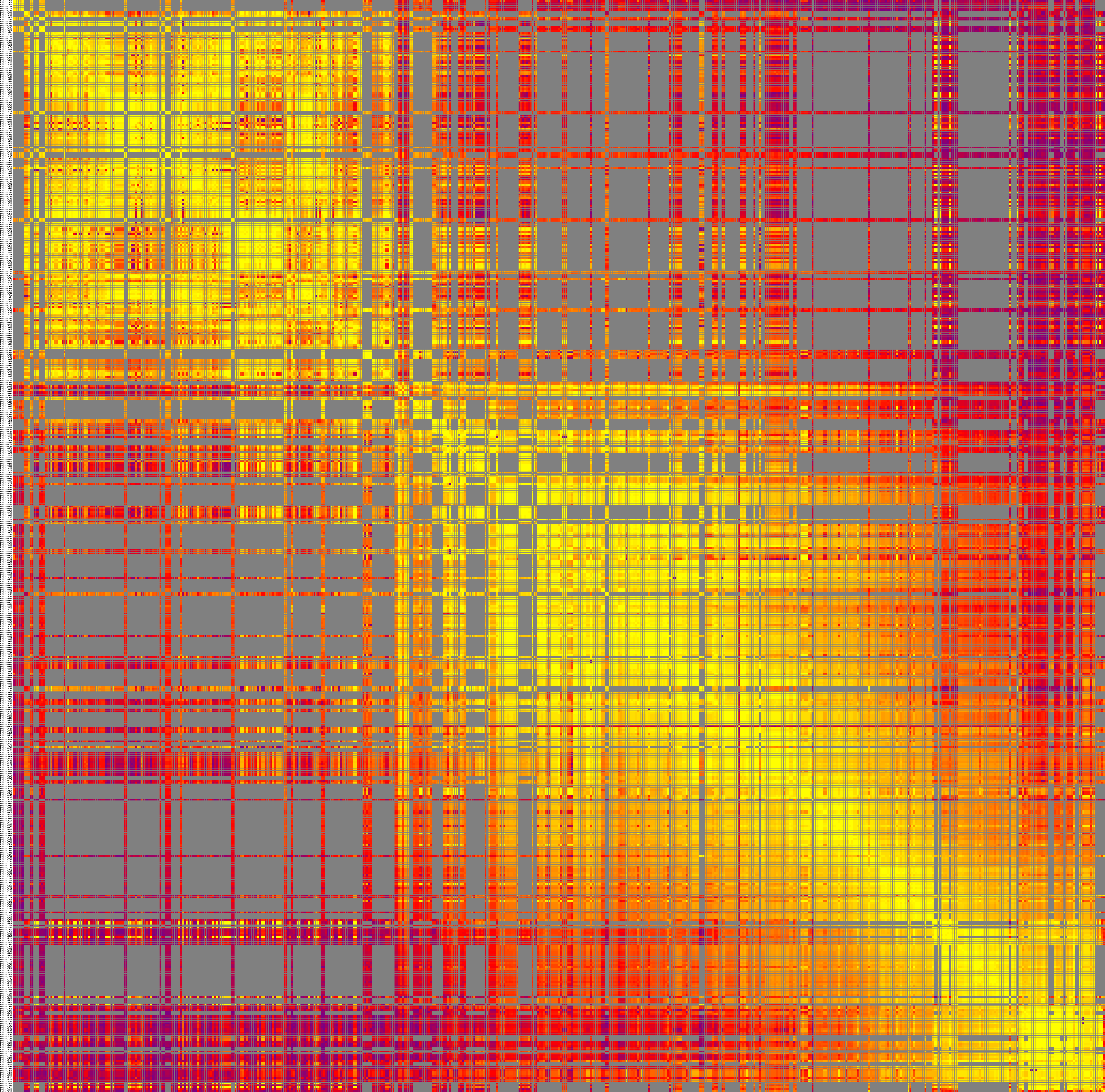

Supplement: S2 File — Each cell represents the recombination rate of two markers. Yellow and purple indicate lower and higher recombination rates, respectively. Gray denotes missing data. (ZIP) [file pone.0181728.s003.zip › S2_File/chr4.sexAver.r.heatMap.png]

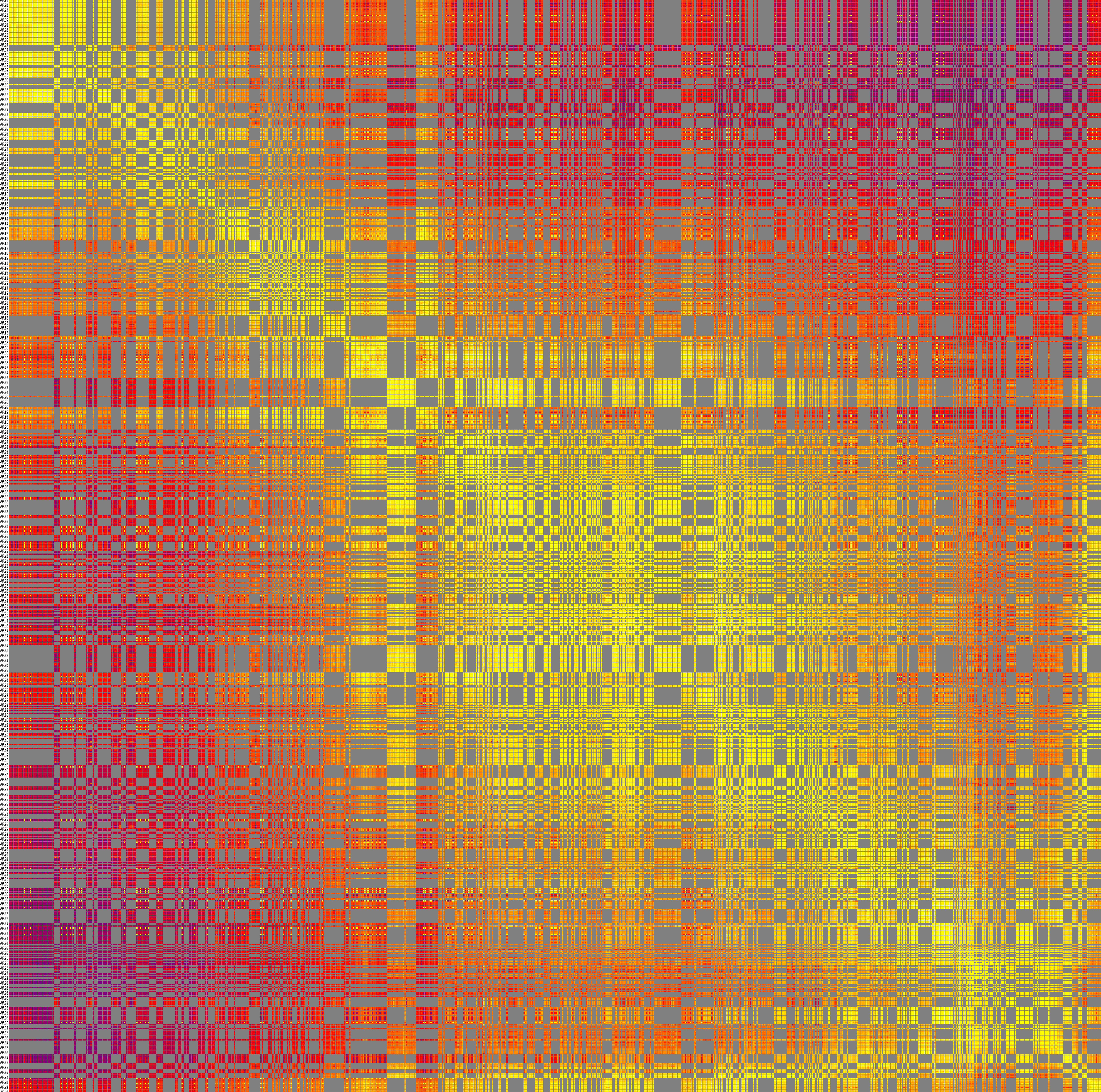

Supplement: S2 File — Each cell represents the recombination rate of two markers. Yellow and purple indicate lower and higher recombination rates, respectively. Gray denotes missing data. (ZIP) [file pone.0181728.s003.zip › S2_File/chr5.sexAver.r.heatMap.png]

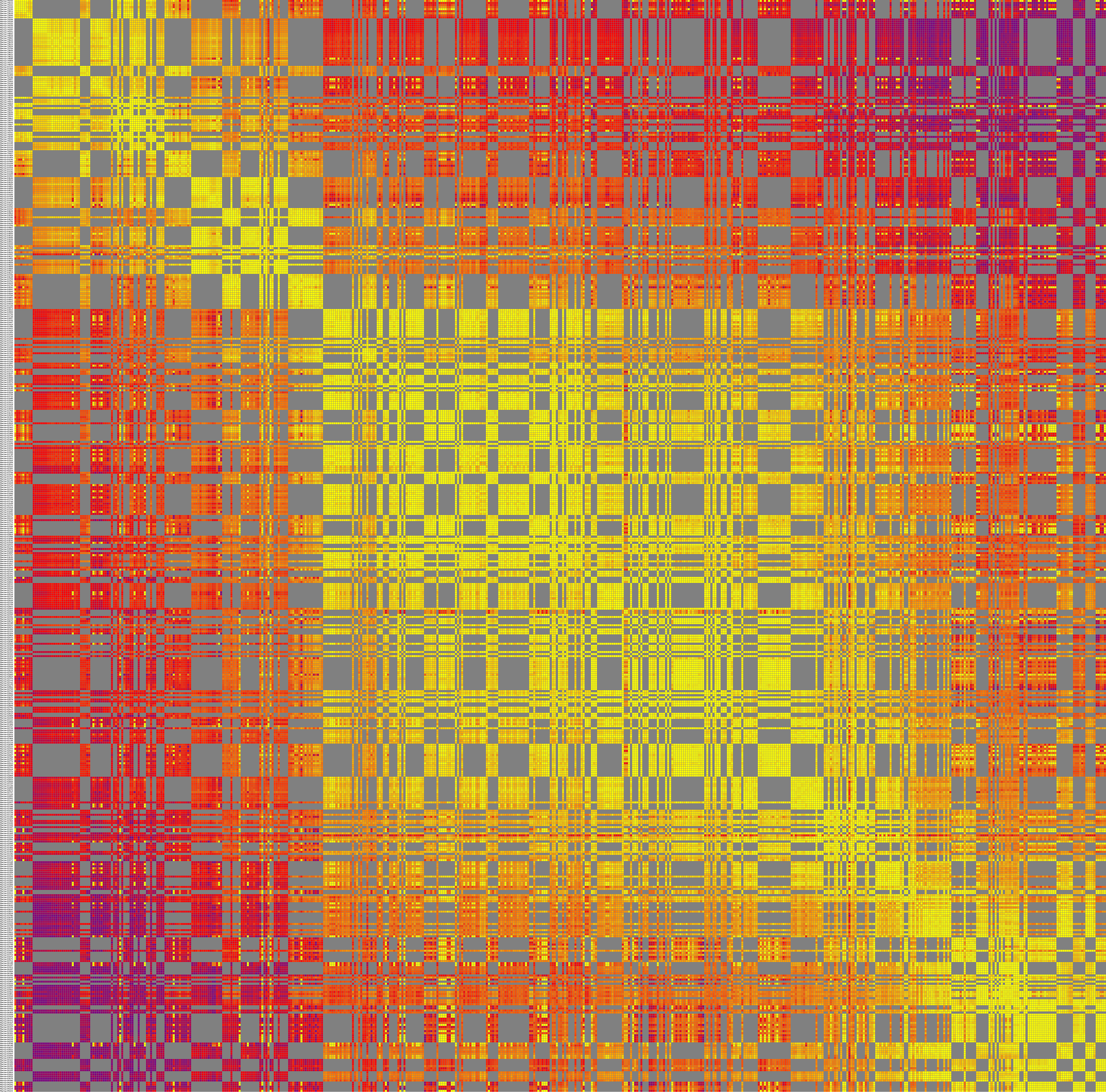

Supplement: S2 File — Each cell represents the recombination rate of two markers. Yellow and purple indicate lower and higher recombination rates, respectively. Gray denotes missing data. (ZIP) [file pone.0181728.s003.zip › S2_File/chr6.sexAver.r.heatMap.png]

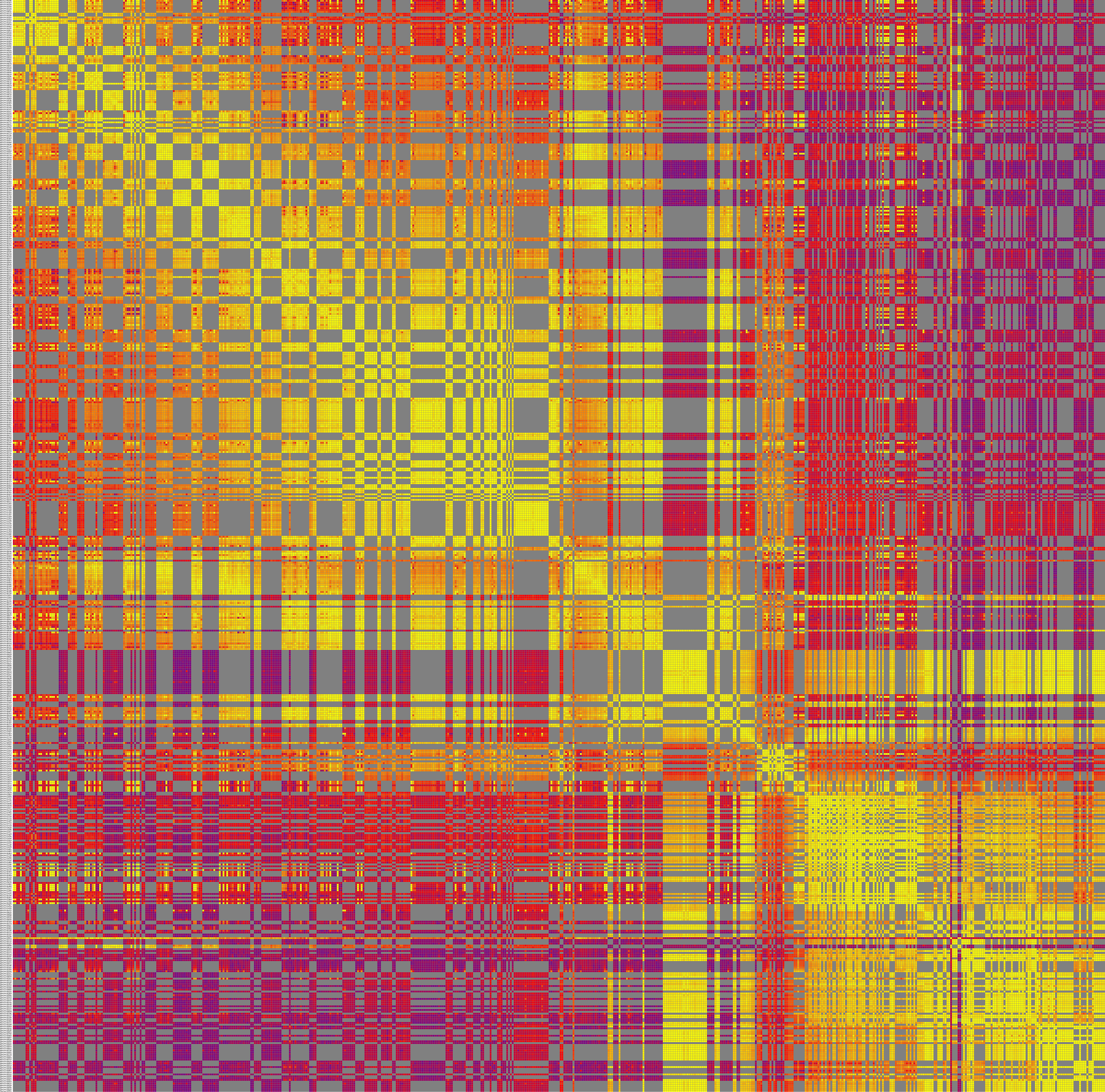

Supplement: S2 File — Each cell represents the recombination rate of two markers. Yellow and purple indicate lower and higher recombination rates, respectively. Gray denotes missing data. (ZIP) [file pone.0181728.s003.zip › S2_File/chr7.sexAver.r.heatMap.png]

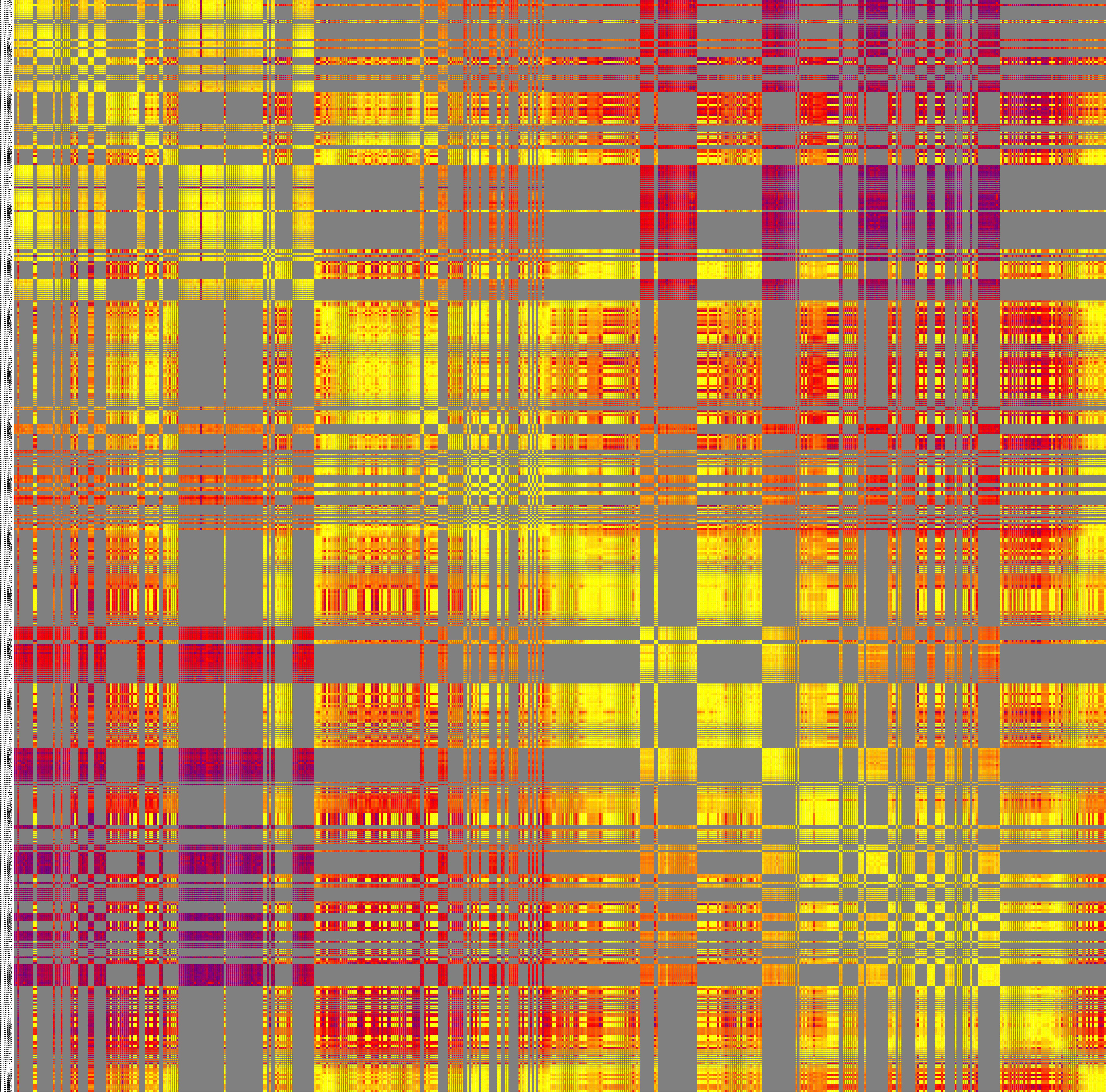

Supplement: S2 File — Each cell represents the recombination rate of two markers. Yellow and purple indicate lower and higher recombination rates, respectively. Gray denotes missing data. (ZIP) [file pone.0181728.s003.zip › S2_File/chr8.sexAver.r.heatMap.png]

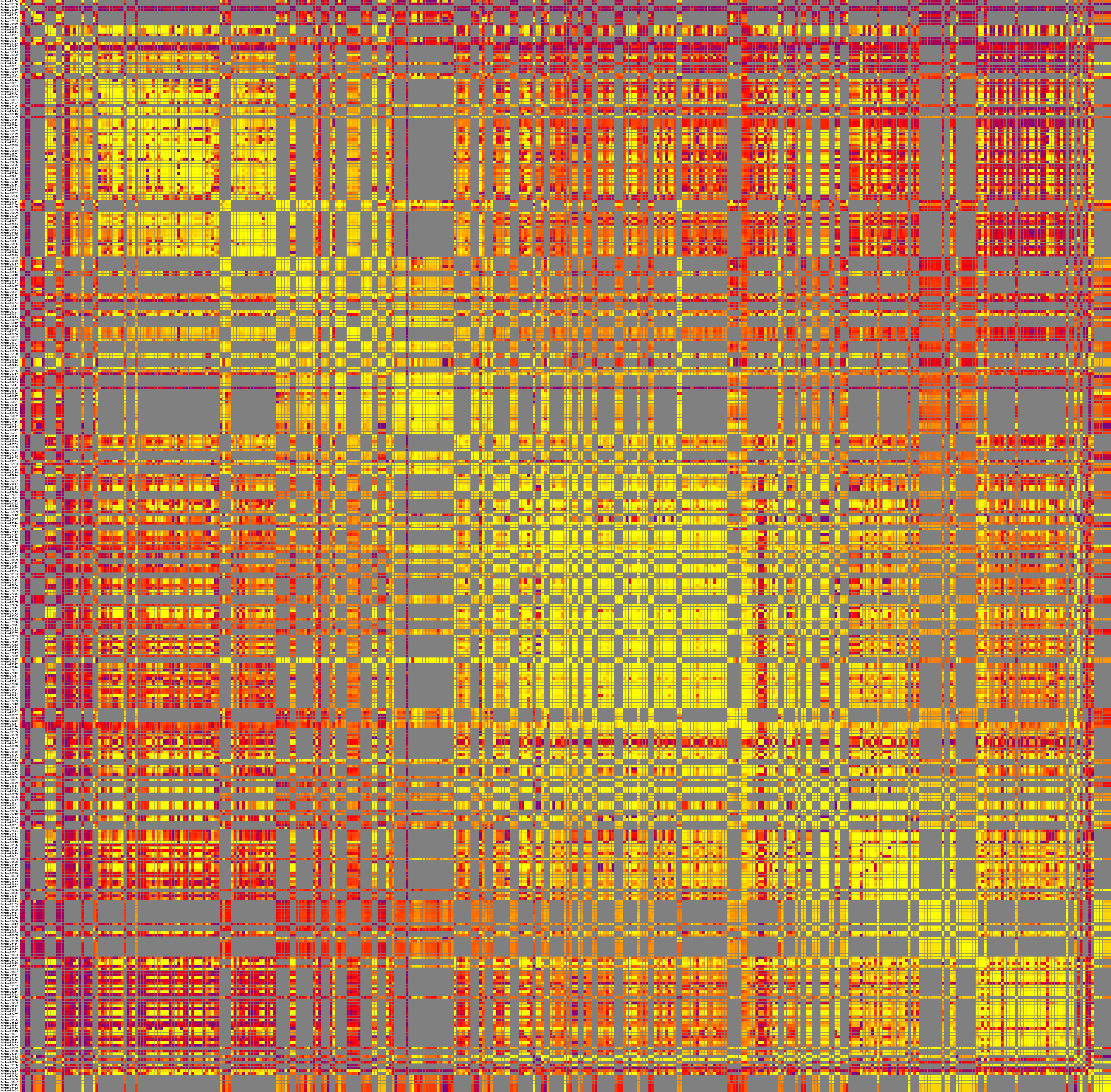

Supplement: S2 File — Each cell represents the recombination rate of two markers. Yellow and purple indicate lower and higher recombination rates, respectively. Gray denotes missing data. (ZIP) [file pone.0181728.s003.zip › S2_File/chr9.sexAver.r.heatMap.png]
